# Humans with inherited MyD88 and IRAK-4 deficiencies are predisposed to hypoxemic COVID-19 pneumonia

**DOI:** 10.1084/jem.20220170

**Published:** 2023-03-03

**Authors:** Ana García-García, Rebeca Pérez de Diego, Carlos Flores, Darawan Rinchai, Jordi Solé-Violán, Àngela Deyà-Martínez, Blanca García-Solis, José M. Lorenzo-Salazar, Elisa Hernández-Brito, Anna-Lisa Lanz, Leen Moens, Giorgia Bucciol, Mohamed Almuqamam, Joseph B. Domachowske, Elena Colino, Juan Luis Santos-Perez, Francisco M. Marco, Claudio Pignata, Aziz Bousfiha, Stuart E. Turvey, Stefanie Bauer, Filomeen Haerynck, Javier Gonzalo Ocejo-Vinyals, Francisco Lendinez, Seraina Prader, Nora Naumann-Bartsch, Jana Pachlopnik Schmid, Catherine M. Biggs, Kyla Hildebrand, Alexandra Dreesman, Miguel Ángel Cárdenes, Fatima Ailal, Ibtihal Benhsaien, Giuliana Giardino, Agueda Molina-Fuentes, Claudia Fortuny, Swetha Madhavarapu, Daniel H. Conway, Carolina Prando, Laire Schidlowski, María Teresa Martínez de Saavedra Álvarez, Rafael Alfaro, Felipe Rodríguez de Castro, ESID Registry Working Party, Gerhard Kindle, Nizar Mahlaoui, Markus G. Seidel, Lougaris Vassilios, Mikko R.J. Seppänen, COVID Human Genetic Effort, Laurent Abel, Alessandro Aiuti, Saleh Al-Muhsen, Fahd Al-Mulla, Mark S. Anderson, Evangelos Andreakos, Andrés A. Arias, Hagit Baris Feldman, Alexandre Belot, Catherine M. Biggs, Dusan Bogunovic, Alexandre Bolze, Anastasiia Bondarenko, Ahmed A. Bousfiha, Petter Brodin, Yenan Bryceson, Carlos D. Bustamante, Manish J. Butte, Giorgio Casari, John Christodoulou, Antonio Condino-Neto, Stefan N. Constantinescu, Megan A. Cooper, Clifton L. Dalgard, Murkesh Desai, Beth A. Drolet, Jamila El Baghdadi, Sara Espinosa-Padilla, Jacques Fellay, Carlos Flores, José Luis Franco, Antoine Froidure, Peter K. Gregersen, Bodo Grimbacher, Filomeen Haerynck, David Hagin, Rabih Halwani, Lennart Hammarström, James R. Heath, Sarah E. Henrickson, Elena W.Y. Hsieh, Eystein Husebye, Kohsuke Imai, Yuval Itan, Erich D. Jarvis, Timokratis Karamitros, Kai Kisand, Cheng-Lung Ku, Yu-Lung Lau, Yun Ling, Carrie L. Lucas, Tom Maniatis, Davood Mansouri, László Maródi, Isabelle Meyts, Joshua D. Milner, Kristina Mironska, Trine H. Mogensen, Tomohiro Morio, Lisa F.P. Ng, Luigi D. Notarangelo, Antonio Novelli, Giuseppe Novelli, Cliona O'Farrelly, Satoshi Okada, Keisuke Okamoto, Tayfun Ozcelik, Qiang Pan-Hammarström, Jean W. Pape, Rebecca Perez de Diego, David S. Perlin, Graziano Pesole, Anna M. Planas, Carolina Prando, Aurora Pujol, Lluis Quintana-Murci, Sathishkumar Ramaswamy, Laurent Renia, Igor Resnick, Carlos Rodríguez-Gallego, Vanessa Sancho-Shimizu, Anna Sediva, Mikko R.J. Seppänan, Mohammed Shahrooei, Anna Shcherbina, Ondrej Slaby, Andrew L. Snow, Pere Soler-Palacín, András N. Spaan, Ivan Tancevski, Stuart G. Tangye, Ahmad Abou Tayoun, Stuart E. Turvey, K M Furkan Uddin, Mohammed J. Uddin, Diederik van de Beek, Donald C. Vinh, Horst von Bernuth, Joost Wauters, Mayana Zatz, Pawel Zawadzki, Helen C. Su, Jean-Laurent Casanova, Isabelle Meyts, Fabian Hauck, Anne Puel, Paul Bastard, Bertrand Boisson, Emmanuelle Jouanguy, Laurent Abel, Aurélie Cobat, Qian Zhang, Jean-Laurent Casanova, Laia Alsina, Carlos Rodríguez-Gallego

**Affiliations:** 1 Pediatric Allergy and Clinical Immunology Dept., Clinical Immunology and Primary Immunodeficiencies Unit, Hospital Sant Joan de Déu, Barcelona, Barcelona, Spain; 2 Study Group for Immune Dysfunction Diseases in Children, Institut de Recerca Sant Joan de Déu, Barcelona, Barcelona, Spain; 3 Clinical Immunology Unit, Hospital Sant Joan de Déu-Hospital Clínic Barcelona, Barcelona, Spain; 4 Laboratory of Immunogenetics of Human Diseases, IdiPAZ Institute for Health Research, La Paz Hospital, Madrid, Spain; 5 Genomics Division, Instituto Tecnológico y de Energías Renovables, Santa Cruz de Tenerife, Spain; 6 Research Unit, Hospital Universitario N.S. de Candelaria, Santa Cruz de Tenerife, Spain; 7 CIBER de Enfermedades Respiratorias, Instituto de Salud Carlos III, Madrid, Spain; 8 Dept. of Clinical Sciences, University Fernando Pessoa Canarias, Las Palmas de Gran Canaria, Spain; 9 St. Giles Laboratory of Human Genetics of Infectious Diseases, Rockefeller Branch, Rockefeller University, New York, NY, USA; 10 Dept. of Intensive Care Medicine, University Hospital of Gran Canaria Dr. Negrin, Canarian Health System, Las Palmas de Gran Canaria, Spain; 11 Dept. of Immunology, University Hospital of Gran Canaria Dr. Negrin, Canarian Health System, Las Palmas de Gran Canaria, Spain; 12 Dept. of Pediatrics, Division of Pediatric Immunology and Rheumatology, Dr. von Hauner Children’s Hospital, University Hospital, Ludwig-Maximilians-Universität München, Munich, Germany; 13 Laboratory for Inborn Errors of Immunity, Dept. of Microbiology, Immunology and Transplantation KU Leuven, Leuven, Belgium; 14 Dept. of Pediatrics, Childhood Immunology, UZ Leuven, Leuven, Belgium; 15 Dept. of Pediatrics, Drexel University College of Medicine, St Christopher’s Hospital for Children, Philadelphia, PA, USA; 16 Dept. of Pediatrics, SUNY Upstate Medical University, Syracuse, NY, USA; 17 Unidad de Enfermedades Infecciosas, Complejo Hospitalario Universitario Insular-Materno Infantil, Las Palmas de Gran Canaria, Spain; 18 Unidad de Gestión Clínica de Pediatría y Cirugía Pediátrica, Hospital Virgen de las Nieves-IBS, Granada, Spain; 19 Dept. of Immunology, Alicante University General Hospital Doctor Balmis, Alicante, Spain; 20 Alicante Institute for Health and Biomedical Research, Alicante, Spain; 21 Dept. of Translational Medical Sciences, Section of Pediatrics, Federico II University, Naples, Italy; 22 Dept. of Pediatric Infectious Diseases and Clinical Immunology, Ibn Rushd University Hospital, Casablanca, Morocco; 23 Clinical Immunology, Autoimmunity and Inflammation Laboratory, Faculty of Medicine and Pharmacy, Hassan II University, Casablanca, Morocco; 24 Dept. of Paediatrics, BC Children’s Hospital, University of British Columbia, Vancouver, Canada; 25 Clinic for Children and Adolescents. Dept. of Hematology and Oncology. University Clinic Erlangen, Erlangen, Germany; 26 Dept. of Pediatric Immunology and Pulmonology, Centre for Primary Immune Deficiency Ghent, Ghent University Hospital, Ghent, Belgium; 27 Dept. of Internal Medicine and Pediatrics, PID Research Laboratory, Ghent University, Ghent, Belgium; 28 Dept. of Immunology, University Hospital Marqués de Valdecilla, IDIVAL, Santander, Spain; 29 Dept. of Pediatric Oncohematology, Hospital Materno Infantil Torrecárdenas, Almería, Spain; 30 Division of Immunology and Children’s Research Center, University Children’s Hospital Zurich, Zurich, Switzerland; University of Zurich, Zurich, Switzerland; 31 Dept. of Pediatrics, CHU St Pierre, ULB/VUB, Brussels, Belgium; 32 Dept. of Internal Medicine, Unit of Infectious Diseases, University Hospital of Gran Canaria Dr. Negrin, Canarian Health System, Las Palmas de Gran Canaria, Spain; 33 Dept. of Immunology, UGC Laboratories, University Hospital of Jaén, Jaén, Spain; 34 Pediatric Infectious Diseases Unit, Hospital Sant Joan de Déu, Barcelona, Spain; 35 CIBER of Epidemiology and Public Health, Madrid, Spain; Translational Research Network in Pediatric Infectious Diseases, Madrid, Spain; 36 Dept. of Surgery and Surgical Specializations, Facultat de Medicina i Ciències de la Salut, University of Barcelona, Barcelona, Spain; 37 Instituto de Pesquisa Pelé Pequeno Príncipe, Faculdades Pequeno Príncipe, Hospital Pequeno Príncipe, Curitiba, Brazil; 38 Dept. of Medical and Surgical Sciences, School of Medicine, University of Las Palmas de Gran Canaria, Las Palmas de Gran Canaria, Spain; 39 Dept. of Respiratory Diseases, University Hospital of Gran Canaria Dr. Negrin, Canarian Health System, Las Palmas de Gran Canaria, Spain; 40 Laboratory of Human Genetics of Infectious Diseases, Necker Branch, INSERM U1163, Necker Hospital for Sick Children, Paris, France; 41 University Paris Cité, Imagine Institute, Paris, France; 42 Pediatric Hematology and Immunology Unit, Department of Pediatrics, Necker Hospital for Sick Children, AP-HP, Paris, France; 43 Department of Pediatrics, Necker Hospital for Sick Children, Paris, France; 44 Howard Hughes Medical Institute, New York, NY, USA

## Abstract

X-linked recessive deficiency of TLR7, a MyD88- and IRAK-4–dependent endosomal ssRNA sensor, impairs SARS-CoV-2 recognition and type I IFN production in plasmacytoid dendritic cells (pDCs), thereby underlying hypoxemic COVID-19 pneumonia with high penetrance. We report 22 unvaccinated patients with autosomal recessive MyD88 or IRAK-4 deficiency infected with SARS-CoV-2 (mean age: 10.9 yr; 2 mo to 24 yr), originating from 17 kindreds from eight countries on three continents. 16 patients were hospitalized: six with moderate, four with severe, and six with critical pneumonia, one of whom died. The risk of hypoxemic pneumonia increased with age. The risk of invasive mechanical ventilation was also much greater than in age-matched controls from the general population (OR: 74.7, 95% CI: 26.8–207.8, P < 0.001). The patients’ susceptibility to SARS-CoV-2 can be attributed to impaired TLR7-dependent type I IFN production by pDCs, which do not sense SARS-CoV-2 correctly. Patients with inherited MyD88 or IRAK-4 deficiency were long thought to be selectively vulnerable to pyogenic bacteria, but also have a high risk of hypoxemic COVID-19 pneumonia.

## Teaser

MyD88- and IRAK-4–deficient patients have a higher risk of hypoxemic COVID-19 pneumonia than individuals of similar age in the general population, due to impaired TLR7-dependent type I IFN production.

## Graphical Abstract

**Figure:**
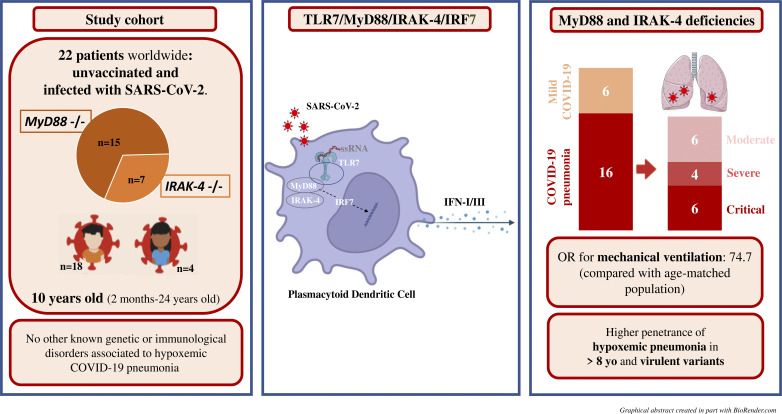

## Introduction

Less than 10% of individuals infected with SARS-CoV-2 develop hypoxemic COVID-19 pneumonia, which may be severe (about 7%) or critical (3%) (Zhang et al., 2022a). Age is the major epidemiological risk factor for hospitalization or death from COVID-19 pneumonia, the risk doubling with every 5 yr of age, from childhood onwards (Zhang et al., 2020a; Knock et al., 2021; Le Vu et al., 2021; O’Driscoll et al., 2021; Sah et al., 2021). The infection fatality rate in unvaccinated individuals is 0.001% at 5 yr of age and 10% at 85 yr of age (a 10,000-fold increase; Bennett et al., 2021; Knock et al., 2021; Le Vu et al., 2021; Navaratnam et al., 2021; O’Driscoll et al., 2021). Most children, adolescents, and young adults with SARS-CoV-2 infection are asymptomatic or present a benign upper respiratory tract disease (Brotons et al., 2021; Chua et al., 2021; Mantovani et al., 2020; Schober et al., 2022; Woodruff et al., 2022). The proportion of asymptomatic infections is greater in children than in adults (Sah et al., 2021). However, interindividual clinical variability remains vast, for all age categories. Various comorbid conditions operate as modest risk factors, with odds ratios (ORs) typically <1.5 and always <2. Men have a 1.5× higher risk of death than women, after correction for other risk factors (Zhang et al., 2020a, 2022a; Brodin, 2021). Likewise, the contribution of common genetic variants detected by genome-wide association studies is modest, the most robustly associated region being a Neanderthal haplotype on chromosome 3 conferring predisposition with an OR around 2 (2.7 in patients <60 yr and 1.5 in patients >60 yr; Nakanishi et al., 2021).

A first molecular explanation for critical COVID-19 pneumonia was provided by inborn errors of TLR3- and/or TLR7-dependent type I IFN immunity, including autosomal recessive (AR) IRF7 and IFNAR1 deficiencies, in about 1–5% of patients, this proportion being lower for individuals over 60 yr of age (Zhang et al., 2020a, 2020b; Asano et al., 2021). This led to the discovery of pre-existing autoantibodies (auto-Abs) against type I IFNs in about 15–20% of patients, with a higher proportion in patients over 70 yr of age (Bastard et al., 2021b, 2022; Bourgeois et al., 2021; Solanich et al., 2021b) and in patients with “breakthrough” hypoxemic COVID-19 pneumonia whose response to RNA vaccines was normal (Bastard et al., 2022). In particular, we found X-linked recessive (XR) TLR7 deficiency in about 1.8% of male patients below the age of 60 yr and in 8.9% of boys (<16 yr) in the COVID Human Genetic Effort (CHGE) consortium cohort (https://www.covidhge.com; Asano et al., 2021; Zhang et al., 2022b). The proportion of patients with combined AR and XR inborn errors of type I IFNs is particularly high in children in this cohort, accounting for 10% of cases of hospitalization for COVID-19 pneumonia (Zhang et al., 2022b). TLR7 is a MyD88/IRAK-4–dependent endosomal receptor for single-stranded RNA in blood plasmacytoid dendritic cells (pDCs), which do not express TLR3 (Asano et al., 2021; Beutler, 2004; Diebold et al., 2004; Reizis, 2019). Conversely, TLR3 is an endosomal receptor of dsRNA in tissue respiratory epithelial cells (RECs), which do not express TLR7 (Zhang et al., 2007a; Guo et al., 2011; Kyung Lim et al., 2019). This genetic approach therefore suggested that both pDCs and tissue respiratory epithelial cells are crucial for type I IFN immunity to SARS-CoV-2 in the respiratory tract (Asano et al., 2021; Casanova and Abel, 2021, 2022; Zhang et al., 2022a).

MyD88 and IRAK-4 are crucial for signaling through the canonical Toll/IL-1 receptor pathway mediated by the 10 human TLRs (including TLR7) other than TLR3, and the IL-1Rs, IL-18R and IL-33R (Beutler, 2004; Kawai and Akira, 2011). Human-inherited MyD88 and IRAK-4 deficiencies are immunological and clinical phenocopies (Alsina et al., 2014; Picard et al., 2010). Affected patients are particularly prone to invasive staphylococcal and pneumococcal bacterial infections in childhood. However, infections become rarer after adolescence (Ku et al., 2007; von Bernuth et al., 2008, 2012, Picard et al., 2010, 2011). Remarkably, due to the abolition of responses driven by TLRs except TLR3, and by all IL-1Rs (in response to all IL-1 paralogs, IL-18, and IL-33), clinical and laboratory signs of inflammation develop slowly in these patients, even during bacterial disease (Picard et al., 2010). Surprisingly, unusually severe viral, fungal, and parasitic diseases have been reported only rarely in patients with MyD88 or IRAK-4 deficiency (Bucciol et al., 2022a; Nishimura et al., 2021; Picard et al., 2010; Tepe et al., 2022; Yang et al., 2005; Zhang et al., 2007a). The only virus reported to cause disease in more than one patient to date is HHV6 (Nishimura et al., 2021; Tepe et al., 2022). The apparent lack of severe viral disease in other known patients is particularly intriguing, as responses to TLR7, TLR8, and TLR9 were abolished in the cells of these patients (Casanova et al., 2011; Yang et al., 2005). Moreover, the four loci encoding the endosomal TLRs sensing nucleic acids—TLR3, TLR7, TLR8, and TLR9—are under stronger negative selection than other TLR loci (Quach et al., 2013).

Indeed, MyD88- and IRAK-4–deficient cells, including fibroblasts and leukocytes, do not produce type I IFN in response to the endosomal TLR7, TLR8, and TLR9 nucleic acid sensors, but they respond normally to TLR3 stimulation (Von Bernuth et al., 2008; Yang et al., 2005). Until recently, the production of type I IFN in response to specific viruses had never been tested for pDCs from patients with MyD88 or IRAK-4 deficiency. pDCs from an IRAK-4–deficient patient were recently shown not to produce type I IFN in response to SARS-CoV-2 (Onodi et al., 2021). Similar findings were obtained for an UNC-93B–deficient patient, whose cells failed to respond to TLR3, TLR7, TLR8, and TLR9 stimulation (Casrouge et al., 2006; Onodi et al., 2021). Nevertheless, the clinical impact of SARS-CoV-2 infection in patients with MyD88 or IRAK-4 deficiency is unclear. Only brief reports have emerged, of three patients in meeting abstracts (Mahmood et al., 2021) or five patients in case reports (Bucciol et al., 2022a; Deyà-Martínez et al., 2021; Goudouris et al., 2021; Milito et al., 2021), describing clinical phenotypes ranging from moderate to critical pneumonia. These patients would be predicted to be prone to hypoxemic COVID-19 pneumonia, due to their complete lack of TLR7-dependent SARS-CoV-2 sensing by pDCs (Asano et al., 2021; Onodi et al., 2021). However, their lack of TLR- and IL-1R–mediated inflammation might, perhaps, mitigate this vulnerability to some extent. We report here the natural course of SARS-CoV-2 infection in these and other patients, for a total of 22 patients from 17 kindreds and eight countries on three continents.

## Results

### Patients with inherited MyD88 or IRAK-4 deficiency

**Figure 1.**
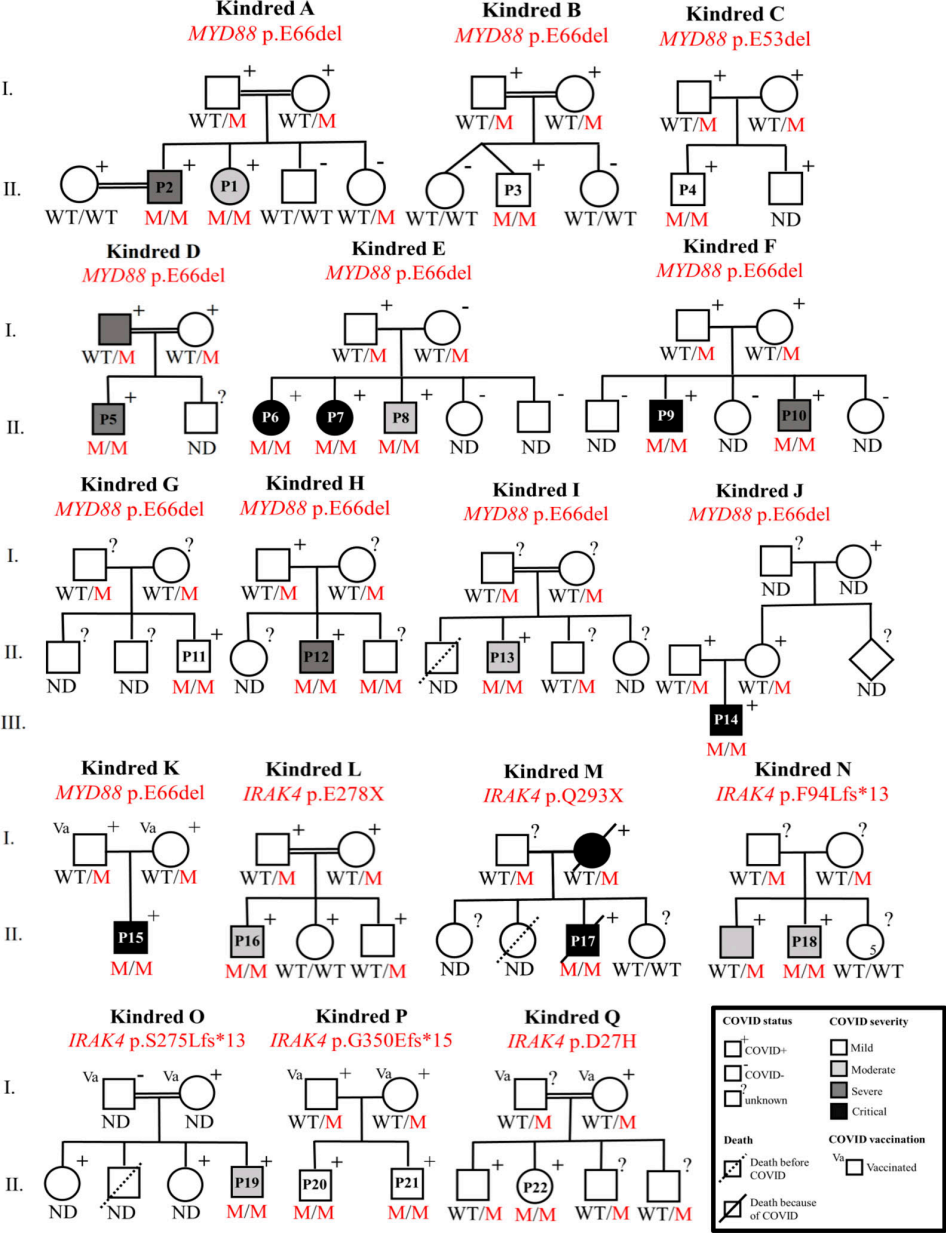
**AR MyD88 and IRAK-4 deficiencies and SARS-CoV-2 infection in 17 kindreds.** Pedigrees of the 17 kindreds containing 7 IRAK-4– and 15 MyD88-deficient patients with SARS-CoV-2 infection (P1–P22 are shown). Patients are identified by the number within the symbol for the individual concerned. The mutations are indicated above each pedigree, and the genotype of each individual is identified below the symbol (M, mutation; ND, no data). Kindred N contains five healthy sisters, indicated by the number “5” within the circle symbol.

Following an international call for collaboration, we obtained data for 22 patients from 17 kindreds with inherited MyD88 (15 patients) or IRAK-4 (7 patients) deficiency, from Spain (7 patients and 6 kindreds), the USA (6 and 3), Belgium (2 and 2), Germany (2 and 2), Canada (2 and 1), Morocco (1), Italy (1), and Switzerland (1), all of whom were infected with SARS-CoV-2 before vaccination (Fig. 1 and Tables 1 and S1). SARS-CoV-2 infection was diagnosed by real-time PCR (RT-PCR; 18 patients) or antigenic assays (3 patients) on nasal swabs after the patients came into contact with a case and/or respiratory clinical manifestations had emerged, or by a serological test in one asymptomatic patient (P11) during routine hospital screening (Table S1). All 10 patients tested were seropositive for SARS-CoV-2–specific IgG/M 4–70 d after infection (Table S1). All but 2 of the 22 patients (the exceptions being P14 and P15) were known to suffer from MyD88 or IRAK-4 or deficiency before the start of the COVID-19 pandemic. All had an AR, complete deficiency, and the genotypes of 10 patients have been reported elsewhere (Yang et al., 2005; Cardenes et al., 2006; von Bernuth et al., 2006, 2008; Ku et al., 2007; Conway et al., 2010; Picard et al., 2010; Weller et al., 2012; Jia et al., 2020; Bucciol et al., 2022a). The *IRAK4* or *MYD88* genotypes of the remaining 12 patients are reported here (Fig. 1). The 22 patients were aged 2 mo to 24 yr (mean: 10.9 ± 6.8 yr). There were 18 (81.8%) male patients and 4 (18.2%) female patients (Fig. 1). 16 patients were receiving prophylaxis at the time of COVID-19 infection: 11 were on oral antibiotics, and 15 were on IgG replacement therapy (IgRT), with 10 patients on both (Table S1). Previous viral infections in these patients are summarized in Tables S2 and S3.

**Table 1.** Demographic and clinical characteristics of MyD88- and IRAK-4–deficient patients with SARS-CoV-2 infection

Kindred/Patient	Age (yr) and gender	Ancestry/country	IEI	Pre-existing comorbidities	Fever^b^ (d/peak)/symptoms	Hospital admission^c^ (d)	ARDS/ICU admission^d^ (d)	Oxygen therapy^e^/days on oxygen therapy (BA)^f^	Other treatments	Severity grade (CHGE score)	Death
A/P1	17 (F)	Roma/Spain	MyD88	Lung calcifications	Yes (9/38.3°C)/Dry cough, odynophagia	Yes (7)	No/No	No/(NA)	Antibiotics, sGC	Moderate	No
A/P2	19 (M)	Roma/Spain	MyD88	Obesity (RF)^a^	Yes (4/38.2°C)/Dry cough, odynophagia	Yes (5)	No/No	cNC (max. 2 Lpm)/(4/3)	Antibiotics, remdesivir, sGC	Severe	No
B/P3	6 (M)	Roma/Spain	MyD88	No	No/Dry cough	No	No/No	No/(NA)	No	Mild/Non-confirmed pneumonia	No
C/P4	1.5 (M)	Roma/Spain	MyD88	No	Yes (1/38°C)/No	Yes (5)	No/No	No/(NA)	Antibiotics	Mild/Non-confirmed pneumonia	No
D/P5	6.5 (M)	Roma/Belgium	MyD88	No	Yes (4/ND)/Cough, rhinorrhea, headache, diarrhea	Yes (9)	No/No	cNC (max. 2 Lpm)/(5/4)	Antibiotics	Severe	No
E/P6	16 (F)	White, not Hispanic/USA	MyD88	Overweight	Yes (8/39.4°C)/ND	Yes (21)	Yes/Yes (19)	iMV/(3/11)	Antibiotics, hydroxychloroquine, sGC, tocilizumab, remdesivir	Critical	No
E/P7	15 (F)	White, not Hispanic/USA	MyD88	Overweight	Yes (5/40.9°C)/ND	Yes (30)	Yes/Yes (28)	iMV, ECMO/(3/13)	Antibiotics, antifungal drugs, remdesivir, hydroxychloroquine, sGC, tocilizumab	Critical	No
E/P8	12 (M)	White, not Hispanic/USA	MyD88	Obesity (RF)	Yes (3/39.0°C)/ND	Yes (7)	No/Yes (5, for observation)	No/(NA)	Antibiotics, hydroxychloroquine	Moderate	No
F/P9	14 (M)	White, not Hispanic/USA	MyD88	Overweight	Yes (5/40.0°C)/ND	Yes (7)	No/Yes (1)	hfNC/(3/5)	Antibiotics, remdesivir, sGC	Critical	No
F/P10	9 (M)	White, not Hispanic/USA	MyD88	No	Yes (3/39.5°C)/ND	Yes (11)	No/No	cNC (max. 3 Lpm)/(6/3)	Antibiotics, remdesivir sGC, baricitinib	Severe	No
G/P11	8 (M)	Roma/Italy	MyD88	No	No/NA	No	No/No	No/(NA)	No	Silent	No
H/P12	8 (M)	Roma/Germany	MyD88	No	Yes (4/39.8 °C)/Cough	Yes (10)	No/No	Indirect oronasal oxygen (max. 3 Lpm)/(2/3)	Antibiotics, sGC, IgRT	Severe	No
I/P13	13 (M)	Roma/Switzerland	MyD88	Epilepsy	Yes (4/39°C)/Cough, sore throat, headache	Yes (4)	No/No	No/(NA)	Antibiotics	Moderate	No
J/P14	1.2 (M)	Roma/Spain	MyD88	No	Yes (10/38.2°C)/ Diarrhea	Yes (25)	Yes/Yes (17)	iMV/(7/16)	Antibiotics, sGC	Critical	No
J/P15	0.17 (M)	Roma/Spain	MyD88	No	Yes (3/38°C)	Yes (17)	Yes/Yes (10)	iMV/(4/13)	Antibiotics, remdesivir, sGC	Critical	No
L/P16	24 (M)	White, not Hispanic/Spain	IRAK4	Eosinophilic esophagitis, allergic rhinosinusitis, nasal polyposis	Yes (7/39°C)/Arthromyalgia, cough, dyspnea	Yes (5)	No/No	No/(NA)	Antibiotics, tenofovir, sGC	Moderate	No
M/P17	23 (M)	White, not Hispanic/USA	IRAK4	Restrictive lung disease Neurocognitive delay (RF)	Yes (94/ND)/Hypothermia (34.8°C on admission)	Yes (94)	Yes/Yes (23)	iMV (family declined ECMO)/ (ND/94)	Antibiotics, remdesivir, sGC	Critical	Yes
N/P18	15 (M)	White, not Hispanic/Morocco	IRAK4	No	No (1/37.5°C)/ND	Yes (3)	No/No	No/(NA)	Antibiotics, hydroxychloroquine	Moderate	No
O/P19	14 (M)	White, not Hispanic/Germany	IRAK4	Neutropenia (700–1,540/mm^3^ baseline)	Yes (3/39.4°C)/Cough, nasal congestion	Yes (4)	No	No/(NA)	Antibiotics, casirivimab + imdevimab	Moderate	No
P/P20	6 (M)	South Asian/Canada	IRAK4	No	Yes (2/38.5°C)	No	No/No	No/(NA)	No	Mild/Non-confirmed pneumonia	No
P/P21	3 (M)	South Asian/Canada	IRAK4	No	Yes (2/38.5°C)	No	No/No	No/(NA)	No	Mild/Non-confirmed pneumonia	No
Q/P22	8 (F)	South Asian/Belgium	IRAK4	No	No	No	No/No	No (NA)	No	Mild/Non-confirmed pneumonia	No

P, patient; M, male, F, female; cNC, conventional nasal cannula; iMV, invasive mechanical ventilation; hfNC, high flow nasal cannula; Lpm, liters per minute; BA, before admission; sGC, systemic glucocorticoids; NA, not applicable; ND, no data.

a RF indicates when the documented comorbidity is a recognized risk factor for severe COVID-19.

b Fever: Yes or no (days of fever/maximum temperature registered).

c Hospital admission: Yes or no (days of hospital stay [including general ward and ICU]).

d Patient with ARDS: Yes or no/Admitted to ICU: Yes or no (total number of days hospitalized in the ICU).

e Oxygen support: Type of oxygen support (higher support) or no.

f (Days from the onset of the first symptoms until the patient needed oxygen therapy/total number of days with oxygen therapy).

### Clinical manifestations of SARS-CoV-2 infection in the patients

**Figure 2.**
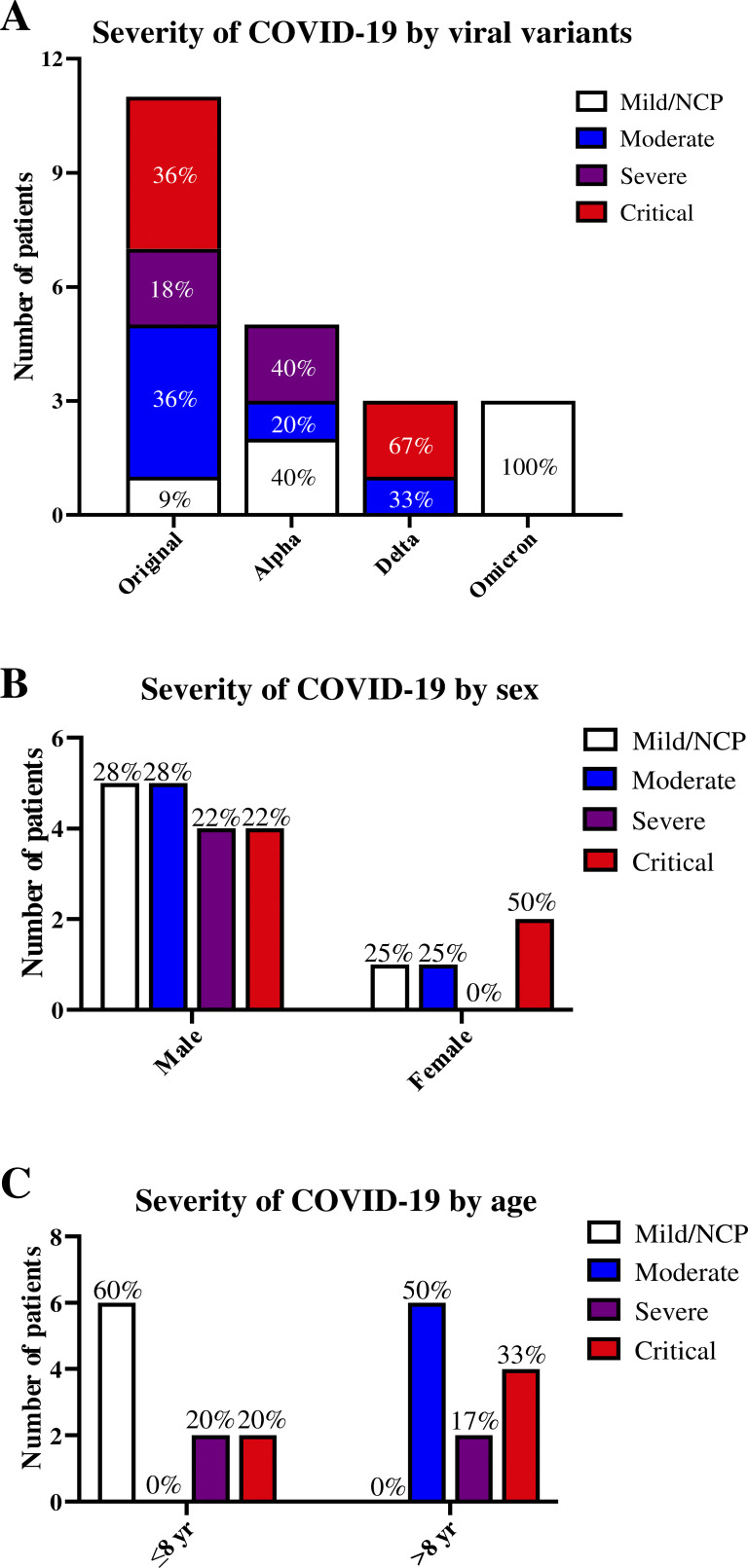
**Severity of SARS-CoV-2 infection in patients with MyD88 or IRAK-4 deficiencies associated with risk factors. (A)** Severity of the infections associated with viral variants. **(B)** Severity of infection as a function of sex. **(C)** Severity of infection as a function of age.

16 of the 22 patients were hospitalized for pneumonia, as confirmed by x ray or computed tomography (CT) scan, including six with moderate pneumonia and 10 with hypoxemic pneumonia, which was critical and required admission to an intensive care unit (ICU) in six cases. Six patients had silent or mild infection, including one with x-ray data (P4, hospitalized as a precaution) and five without x-ray or CT scan data. None of the patients infected with the Omicron variant suffered from pneumonia, whereas patients infected with other viral variants had a wide spectrum of clinical manifestations, ranging from silent infection to death, indicating that the viral variant involved had a major impact on infection severity (Fig. 2 A). RT-PCR cycle threshold (Ct) values were available for 10 patients and were highly variable, even in patients with similar disease severity (Table S1). This is not surprising, because Ct values are dependent on both the RT-PCR platform and the RT-PCR assay used, and on the SARS-CoV-2 genomic variant (Amorim et al., 2022; Fomenko et al., 2022; Kogoj et al., 2022; Mohsin and Mahmud, 2022; Ong et al., 2022). Sex had no major impact on the severity of infection, as 44.4% of male patients and 50% of female patients developed hypoxemic pneumonia (Fig. 2 B). By contrast, age had a major effect on disease severity in patients with MyD88/IRAK-4 deficiency. Indeed, 60% of patients under the age of 8 yr had mild infections, without pneumonia, whereas all patients over the age of 8 yr had pneumonia, which was hypoxemic in 50% of cases, with one death (P17, 23 yr; Fig. 2 C). The chances of developing hypoxemic pneumonia were, therefore, much higher in older patients. Thus, the risk factors for the general population, including viral variant and age, also had a detectable impact on the severity of COVID-19 pneumonia in MyD88- or IRAK-4–deficient patients. The penetrance of hypoxemic pneumonia was higher in older patients infected with more virulent variants than in younger patients infected with the Omicron variant.

### Clinical course of disease in the 16 patients with COVID-19 pneumonia

The time from first symptoms to hospital admission for these 16 patients was 3.3 ± 1.7 d. For the 12 patients for whom the mode of transmission was known (Table S1), the time from first contact with an individual with confirmed SARS-CoV-2 infection to hospitalization was 8.3 ± 4.7 d. The mean duration of hospitalization for patients with moderate, severe, or critical pneumonia was 5.0 (range: 3–7), 8.8 (range: 5–11), and 31.3 (range: 7–94) d, respectively. The six patients with critical pneumonia had a mean duration of ICU stay of 16.3 d (range: 1–28 d; Table 1). Mean time from first symptoms to oxygen therapy in our 10 patients with hypoxemic pneumonia was 4.0 (range: 2–7) d, and the patients were on oxygen therapy for 16.2 (range: 3–94) d (3.3 d, range: 3–4 d for severe pneumonia vs. 24.8 d, range: 5–94 d for critical pneumonia, P = 0.013). The 16 patients with confirmed pneumonia received antibiotics; 11 patients received systemic glucocorticoids, 7 received remdesivir, 1 received tenofovir, 4 received hydroxychloroquine, 2 received tocilizumab, 1 received baricitinib, and P19, who had moderate pneumonia, was treated with SARS-CoV-2–neutralizing mAbs (casirivimab plus imdevimab), 2 d after the onset of symptoms. One of the 22 patients (P17, 23 yr old) died (Table 1). The course of SARS-CoV-2 infection in patients with pneumonia was generally severe, albeit with some interindividual variability.

### Lack of other known genetic or immunological disorders in the patients

We screened the patients for other genetic or immunological disorders known to cause hypoxemic COVID-19 pneumonia (Bastard et al., 2020, 2021b, Zhang et al., 2020b, 2022b; Asano et al., 2021). Autoantibodies neutralizing IFN-α2, IFN-β, and IFN-ω were not detected in any of the 12 patients tested, 3 of whom had mild infections and 9 of whom had COVID-19 pneumonia (5 moderate, 2 severe, and 2 critical). We also sequenced the exomes of 13 patients and analyzed the available exomes of 2 other patients. We screened our 15 patients for rare (MAF < 10^−3^ according to the gnomAD database) predicted loss-of-function (pLOF) variants of the 478 genes known to underlie AR, autosomal dominant (AD), or XR inborn errors of immunity (IEIs; Tangye et al., 2022). Among the five patients with mild SARS-CoV-2 infection, three patients carried candidate variants potentially linked to an IEI (Table S4). Noteworthy, two brothers with mild COVID-19 (P20 and P21) carried a heterozygous variant (p.P301L) in the *OAS1* gene. AR LOF mutations in OAS1 were recently shown to cause the multisystem inflammatory syndrome in children (Lee et al., 2022); and heterozygous *OAS1* gain-of-function (GOF) variants cause a polymorphic autoinflammatory immunodeficiency (OPAID), characterized by recurrent fever, dermatitis, inflammatory bowel disease, pulmonary alveolar proteinosis, and hypogammaglobulinemia (Cho et al., 2018; Magg et al., 2021). Both patients and their parents (fully vaccinated against SARS-CoV-2) developed a mild SARS-CoV-2 infection and did not have any of the clinical or immunological signs of gain-of-function– or LOF-OAS1 deficiency.

Among 10 patients with COVID-19 pneumonia (5 moderate, 2 severe, and 3 critical), 4 patients carried variants that could potentially be linked to an IEI (Table S4). Two patients, P14 (critical pneumonia) and P18 (moderate pneumonia), carried heterozygous variants (p.R756W and p.E941K, respectively) in *RTEL1*. AR and, more rarely, AD mutations in *RTEL1* are associated with dyskeratosis congenita, and AD variants in *RTEL1* have also been associated with idiopathic pulmonary fibrosis, even with a late or very late onset (Borie et al., 2019; Moore et al., 2019; Newton et al., 2022; Stuart et al., 2015; Walne et al., 2013). Neither the patients nor their parents had a history of any of the cardinal features of dyskeratosis congenita or of idiopathic pulmonary fibrosis, although no CT scans were performed. Heterozygous variants in *POL3RA* and *IFNAR1* were found in P15 (critical pneumonia). The variant in *POLR3A* found in P15 is the same as that observed in P3 and his mother, both unvaccinated against SARS-CoV-2 and with mild COVID-19 (Table S4 and Fig. 1) and no previous varicella-zoster virus infection or any pathological predisposition to viral infectious diseases. AD mutations in *IFNAR1*, which encodes a subunit of the type I IFN receptor, have been previously shown to cause critical COVID-19 pneumonia (Zhang et al., 2020b). The impact on expression and function of the observed p.Q80H mutation in *IFNAR1* was studied and found to be neutral (Zhang et al., 2020b). Finally, P17 (critical pneumonia) was found to be homozygous for a p.E941K variant in *APOL1*. Many African individuals express *APOL1* variants that, in heterozygosity, counteract resistance factors from human infective trypanosomes, enabling them to avoid sleeping sickness. In addition, the *APOL1* variants that confer protection against trypanosomiasis are associated with chronic kidney disease, particularly in the context of virus-induced inflammation such as COVID-19 (Pays et al., 2022; Zhang et al., 2019). It is unlikely that these variants predisposed to critical COVID-19 pneumonia in our patient with no documented chronic kidney disease and normal serum creatinine levels in the course of his SARS-CoV-2 infection.

None of the patients carried candidate variants at any IEI loci (Tangye et al., 2022), suggesting that other known genetic disorders did not contribute to their poor control of SARS-CoV-2. Thus, we did not detect auto-Abs against type I IFN or additional genetic defects in the patients. However, these findings do not exclude the possibility that other genetic or acquired modifiers affected the outcome of SARS-CoV-2 infection in these patients. Collectively, however, they suggest that the *MYD88* and *IRAK4* genotypes were the main drivers of COVID-19 pneumonia in 15 of the 22 patients.

### More severe COVID-19 pneumonia in patients with MyD88/IRAK-4 deficiencies than in age-matched individuals from the general population

**Figure 3.**
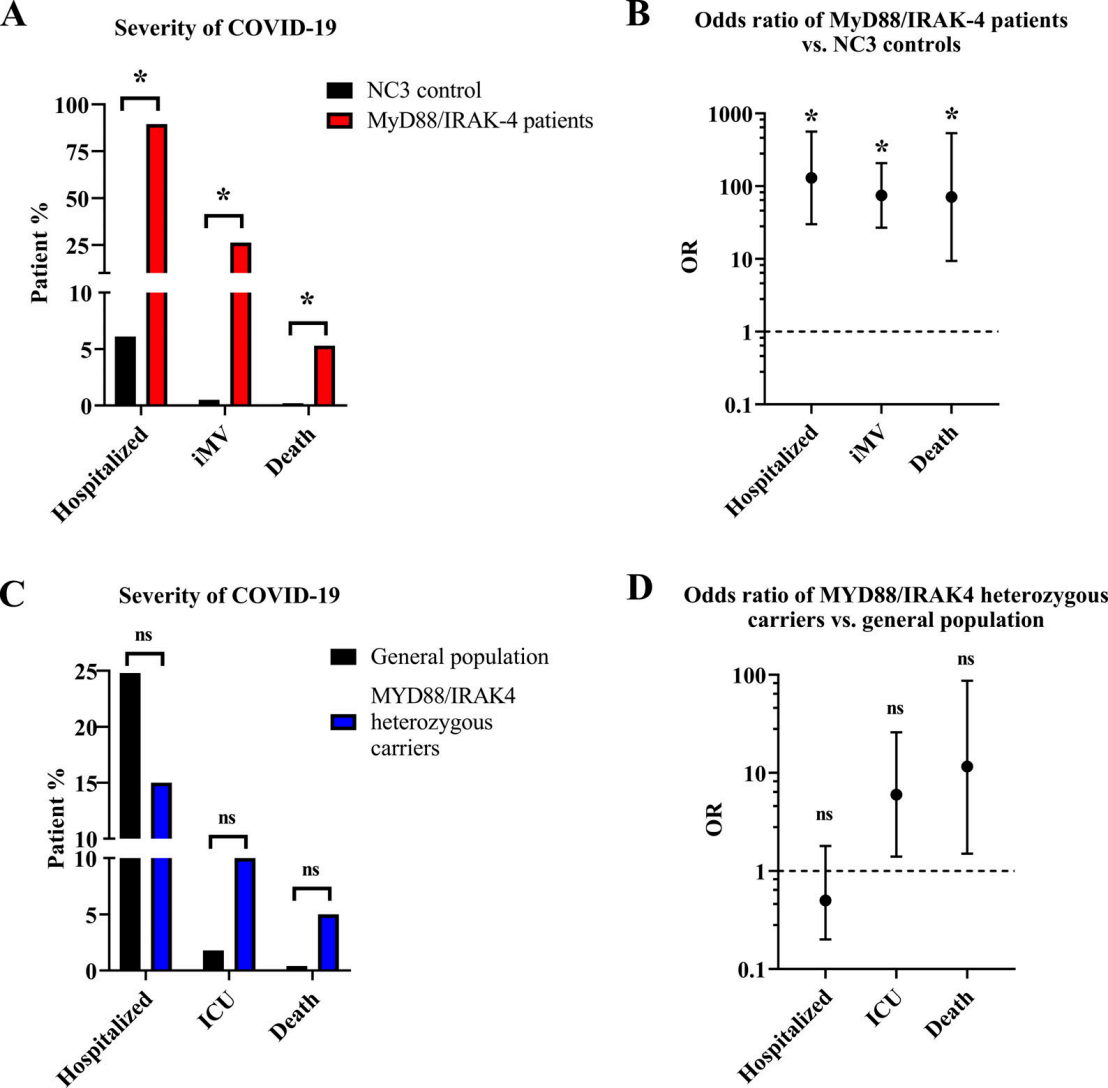
**Susceptibility to severe COVID-19 in patients with MyD88 or IRAK-4 deficiency. (A and B)** Severity (A) and OR (B) of SARS-CoV-2 infection in MyD88/IRAK-4–deficient patients relative to the age-matched controls from the NC3 cohort infected with the same viral variants. *, P < 0.001. **(C and D)** Severity (C) and OR (D) of SARS-CoV-2 infection in heterozygous relatives of MyD88- or IRAK-4–deficient patients relative to the Spanish general population between the ages of 20 and 49 yr after the first wave (April 2020).

We compared the risks of hospitalization and critical COVID-19 pneumonia between our patients and a retrospective series of 167,262 SARS-CoV-2–infected children before the emergence of the Omicron variant (mean age: 11.9 yr; interquartile range [IQR]: 6.0–16.1 yr) within the National COVID Cohort Collaborative (NC3) cohort (total 1,068,410 children <19 yr of age, NC3, USA). We included only 19 patients from our cohort who were also infected with the original and Delta variants. These two cohorts had similar age distributions (mean age of patients with MyD88 or IRAK-4 deficiency: 13.0 yr [IQR 6.5–16.0] vs. NC3 series: 11.9 yr [IQR 6–16.1]), but there were significantly more male patients in our cohort (84.2%) than in the NC3 cohort (50.1%; P = 0.006; OR 5.3, 95% confidence interval [CI] 1.5–18.1). In total, 10,245 (6.1%) of the controls were hospitalized (Martin et al., 2022a). The risk of hospitalization was significantly higher in MyD88- or IRAK-4–deficient patients (89.5%) than in the N3C series (6.1%; P < 0.001; OR: 130.3; 95% CI: 30.1–563.9; Fig. 3, A and B). Invasive mechanical ventilation was required for 796 of the 167,262 patients positive for SARS-CoV-2 in the NC3 series (0.5%), and for 5 of our 19 patients (26.3%; P < 0.001; OR: 74.7, 95% CI: 26.8–207.8). Moreover, the infection fatality rate was significantly higher in our patients (5%) than in the NC3 series (0.08%; P < 0.001; OR: 70.9, 95% CI: 9.4–535.2; Fig. 3, A and B). Overall, the clinical manifestations of SARS-CoV-2 infection were much more severe in MyD88- or IRAK-4–deficient patients than in age-matched individuals from the general population.

### Similar COVID-19 pneumonia severity in patients with MyD88/IRAK-4 deficiencies and patients with TLR7 deficiency

35 male patients with SARS-CoV-2 infection and experimentally confirmed TLR7 deficiency have been reported to date (van der Made et al., 2020; Asano et al., 2021; Fallerini et al., 2021; Solanich et al., 2021a; Zhang et al., 2022b). We compared 22 patients with MyD88/IRAK-4 deficiency with 35 patients with TLR7 deficiency. Globally, MyD88/IRAK-4–deficient patients were less likely to develop severe infections (mild infections: 27.3 vs. 5.7%; moderate: 27.3 vs. 2.9%; severe: 18.2 vs. 11.4%; critical: 27.3 vs. 80%; P = 0.0002). These differences cannot be explained by the sex of the patients because they remained significant when only the 18 male patients with MyD88 or IRAK-4 deficiency were considered (P = 0.0001). There may have been an ascertainment bias, because the IRAK-4– and MyD88-deficient patients in our cohort were recruited prospectively, whereas most of the TLR7-deficient patients were recruited retrospectively. We, therefore, also compared SARS-CoV-2 infection severity between our cohort and a subgroup of seven prospectively recruited TLR7-deficient patients (Asano et al., 2021; Solanich et al., 2021a; Zhang et al., 2022b). No significant differences in age were found between our 22 patients (mean 10.9 ± 6.8 yr) and these 7 TLR7-deficient patients (25.4 ± 16.9; P *=* 0.06). No statistically significant differences in disease severity were observed either (mild: 27.3 vs. 28.6%; moderate: 27.3 vs. 14.3%; severe: 18.2 vs. 14.3%; critical: 27.3 vs. 42.9%; P = 0.9). However, the group of prospectively recruited TLR7-deficient patients was small. Overall, COVID-19 severity appears to be similar in patients with MyD88/IRAK-4 deficiency and in those with TLR7 deficiency. The difference between these groups is clearly less pronounced than that between either group of patients and age-matched individuals from the general population.

### Lack of MYD88 or IRAK4 co-dominance for COVID-19 pneumonia

The bacterial infection phenotype of MyD88 or IRAK-4 deficiency is clearly recessive (Picard et al., 2010), but we analyzed possible co-dominance for the COVID-19 phenotype in 20 unvaccinated household relatives heterozygous for *MYD88* or *IRAK4* (mean age: 32.4 yr, SD: 12.3 yr, range: 10–52 yr). Given their age distribution and the Spanish origin of 6 of the 15 kindreds, we compared the hospitalization rate, ICU need, and mortality of this cohort with data for the Spanish general population between the ages of 20 and 49 yr after the first wave in April 2020 (Cannistraci et al., 2021; Ministerio de Sanidad, 2022). We found no significant differences in hospitalization rate (3 of 20, 15%, of MyD88/IRAK-4 heterozygous relatives vs. 8,823 of 35,583 (24.8%) in the general population, P *=* 0.4; OR: 0.5, 95% CI: 0.2–1.8), ICU admission (2/20–10.0% vs. 647/35,583 [1.8%], P *=* 0.06; OR: 6.0, 95% CI: 1.4–25.9) or mortality (1/20 [5.0%] vs. 161/35,583 [0.4%]; P *=* 0.2; OR: 11.6, 95% CI: 1.5–87.0; Fig. 3, C and D). We also searched for an enrichment in rare (gnomAD frequency <10^−3^) pLOF variants of IRAK4 and MYD88 in 3,269 patients with critical COVID-19, and 1,373 controls with asymptomatic or mild SARS-CoV-2 infection from the CHGE (Matuozzo et al., 2022). We identified three heterozygous individuals among the patients with critical disease and one among the controls with mild disease (P = 0.09). Overall, our results suggest that heterozygous carriers may not have a higher risk of hypoxemic COVID-19, or that penetrance is very low in these individuals.

### Systemic inflammation during SARS-CoV-2 infection

**Figure 4.**
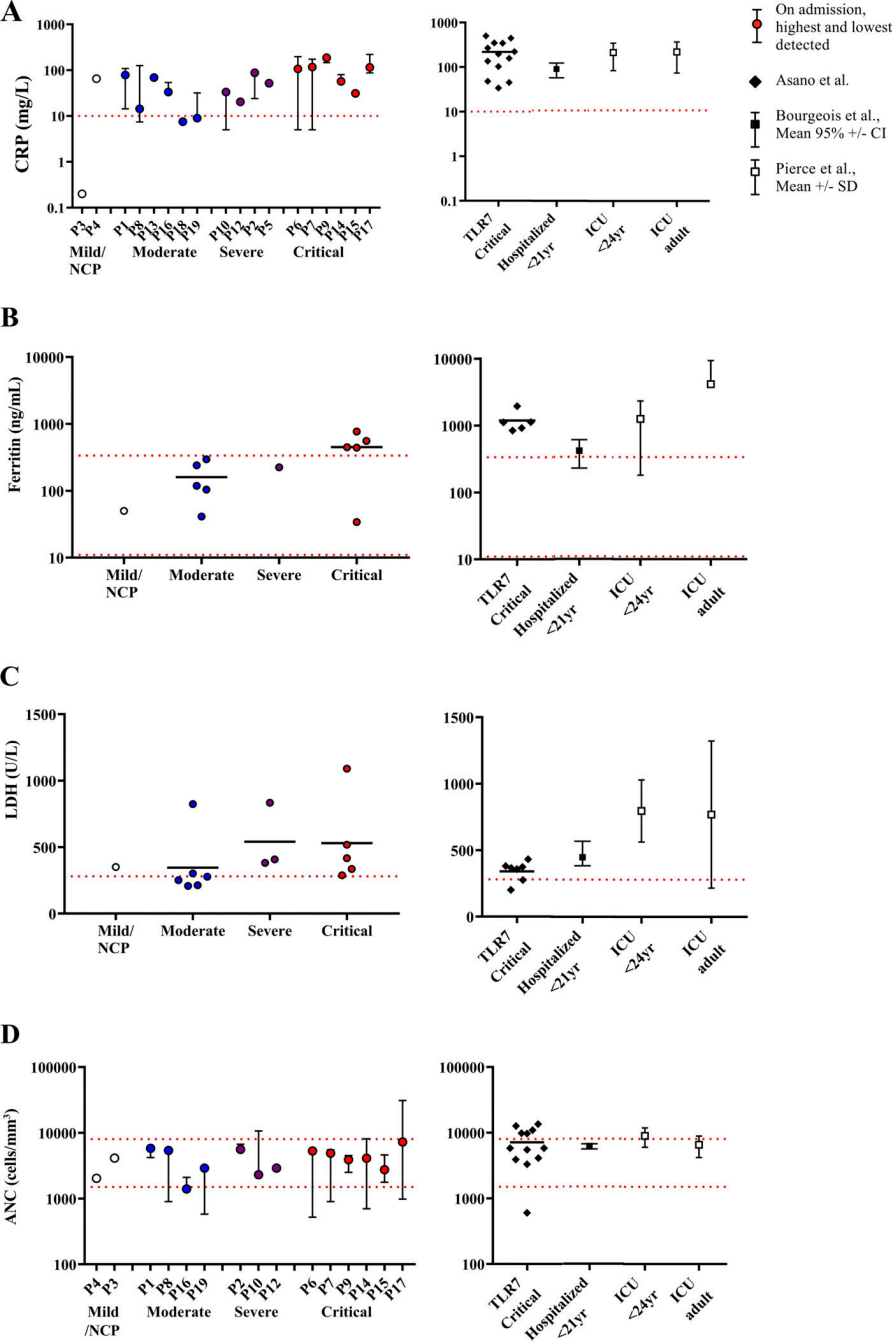
**Inflammation markers in MyD88/IRAK-4-deficient patients during acute infections. (A)** Left: CRP on admission (colored dots), highest level detected (upper bar), and lowest level detected (lower bar) in hospitalized patients with MyD88 or IRAK-4 deficiency. Right: CRP on admission in patients with TLR7 deficiency, hospitalized members of the general population <21 yr of age (Bourgeois et al., 2021), or members of the general population admitted to the ICU (Pierce et al., 2020). Dashed red line: normal range of CRP concentration (<10 mg/dl). **(B and C)** Ferritin (B) and LDH (C) concentrations in patients with MyD88 or IRAK-4 deficiency (left panels), or TLR7 deficiency, hospitalized members of the general population <21 yr of age (Bourgeois et al., 2021), or members of the general population admitted to the ICU (Pierce et al., 2020; right panels). Dashed red lines: normal range of ferritin (11–336 ng/ml) and LDH (<280 U/liter) concentrations. **(D)** Left: ANC on admission (colored dots), highest level detected (upper bar), and lowest level detected (lower bar) in hospitalized patients with MyD88 or IRAK-4 deficiency. Right: ANC on admission in patients with TLR7 deficiency, hospitalized members of the general population <21 yr of age (Bourgeois et al., 2021), or members of the general population admitted to the ICU (Pierce et al., 2020). Dashed red line: normal range of ANC (1,500–8,000 cells/mm^3^).

In 7 of the 16 hospitalized patients for whom data were available, hypothermia (1 patient) or low-grade fever (37.5–38.3°C; 6 patients) was documented; the other 9 patients developed a fever with a body temperature between 39 and 40.9°C. Thus, most patients were able to mount a fever, contrasting with the poor or delayed fever mounted in response to pyogenic bacteria in such patients (Picard et al., 2010). We also studied the blood levels of inflammatory markers including C reactive protein (CRP), ferritin, lactate dehydrogenase (LDH), and absolute neutrophil count (ANC), in hospitalized patients upon admission, and the highest or lowest levels detected if multiple measurements were collected. Overall, most of the patients had high CRP, ferritin, and LDH levels (Fig. 4, A–C). The levels of ferritin and LDH seemed to be particularly high in the patients with the most severe disease, indicating a strong inflammatory response, at odds with previous studies of inflammation in the course of bacterial disease in these patients (Picard et al., 2003; von Bernuth et al., 2008; Picard et al., 2010). However, MyD88- or IRAK-4–deficient patients with critical COVID-19 had somewhat lower blood CRP, ferritin, and LDH levels than TLR7-deficient patients with critical COVID-19 pneumonia (Asano et al., 2021), hospitalized patients under the age of 21 yr from the general population (Bourgeois et al., 2021), and patients from the general population admitted to the ICU (Pierce et al., 2020), although this difference was not statistically significant (Fig. 4, A–C). High ANC is another marker of systemic inflammation often observed in patients with severe COVID-19 pneumonia (Pierce et al., 2020; Bennett et al., 2021; Martin et al., 2022a). However, neutropenia is rare in the acute phase of infection, even in critical cases (Manson et al., 2020; Pierce et al., 2020; Bennett et al., 2021; Bourgeois et al., 2021; Martin et al., 2022a). Surprisingly, we observed frequent neutropenia (ANC < 1,500/mm^3^) in MyD88- or IRAK-4–deficient patients (Fig. 4 D). Seven of the hospitalized MyD88- or IRAK-4–deficient patients developed neutropenia during the acute phase of infection (three of four moderate cases and four of six critical cases), whereas only two had a high ANC at a particular time point (ANC > 8,000/mm^3^; Fig. 4 D and Table S5). By contrast, only 1 of 12 TLR-7–deficient patients with critical disease developed neutropenia and 5 had a high ANC (Fig. 4 D). This phenotype was also observed in MyD88- and IRAK-4–deficient patients with pyogenic bacterial infections (Picard et al., 2003; Picard et al., 2010). Overall, IRAK-4– and MyD88-deficient patients were able to mount an inflammatory response to SARS-CoV-2 infection, with characteristic neutropenia potentially due to defective IL-1R signaling.

### Blood transcriptome inflammatory signature in patients during COVID-19

**Figure 5.**
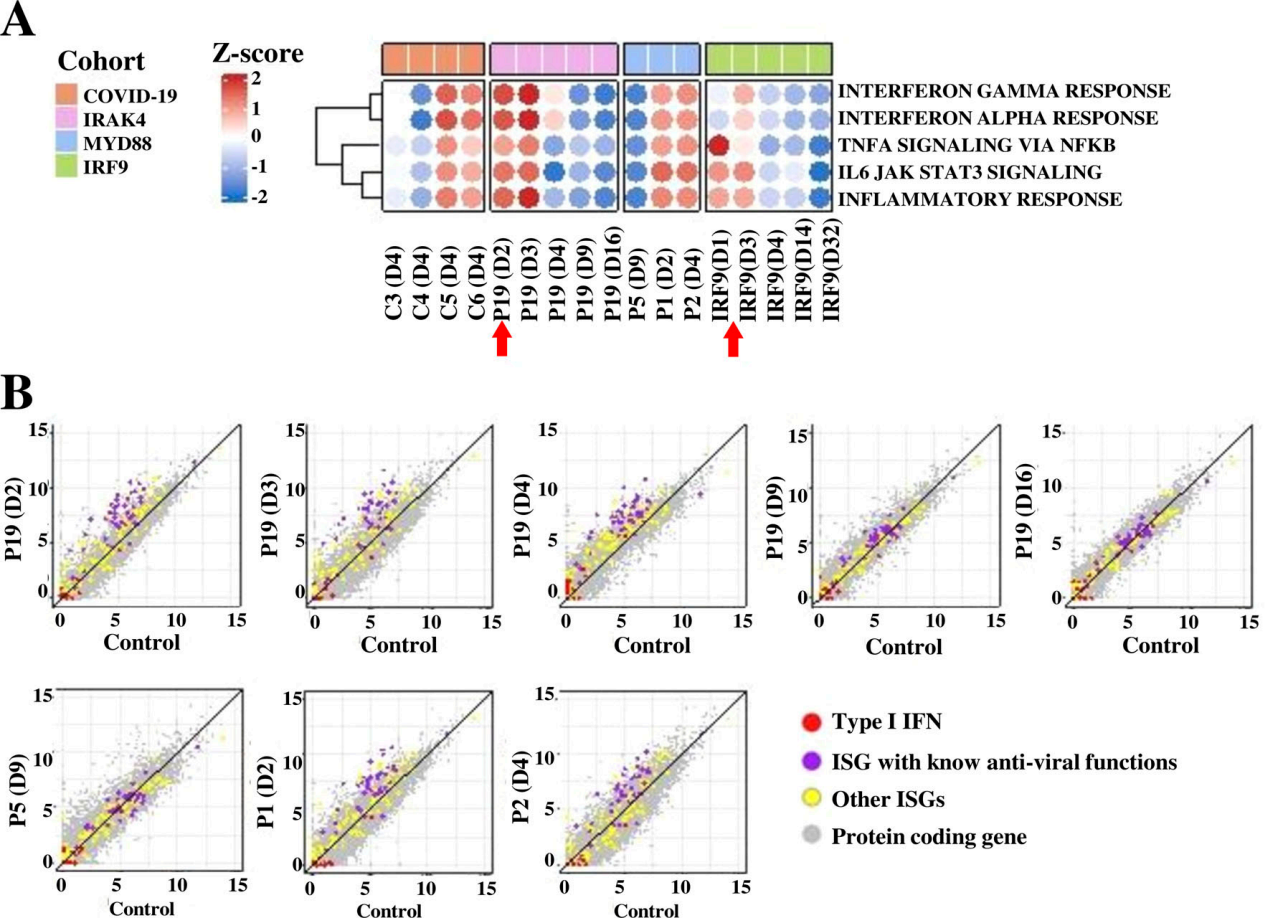
**Transcriptome analysis of whole-blood samples from SARS-CoV-2–infected individuals. (A)** Single-sample gene set enrichment analysis (Hänzelmann et al., 2013) was used to evaluate the IFN-α response, the IFN-γ response, TNF-α signaling through NF-κB, IL-6 JAK-STAT3 signaling, and the inflammatory response. There was one sample for each time-point and patient, and the assay was performed once for each sample. Dot heatmap representing pathway enrichment scores for individual samples. The enrichment score is represented by a colored spot, with red indicating an increase in abundance and blue indicating a decrease in abundance. The intensity of the spots reflects the enrichment score. **(B)** Time-dependent consistent changes in transcript abundance for type I IFN (red), ISGs with known antiviral functions (purple), other ISGs (yellow), and protein-coding genes (gray) are represented on a scatter plot for IRAK4- and MyD88-deficient patients and a non-infected healthy control. In parentheses, D indicates the number of days after positive RT-PCR for controls and the days after symptom onset for patients. Red arrows indicate the day of treatment with mAbs (casirivimab and imdevimab).

We also performed a transcriptome analysis focused on the genes of the inflammatory response to infection. The four patients studied had COVID-19 pneumonia (two moderate and two severe cases). The inflammatory response has been shown to be correlated with COVID-19 severity in several studies (Bennett et al., 2021; Kim and Shin, 2021; Maleknia et al., 2022; Martin et al., 2022a). In our patients, the upregulation of genes involved in the inflammatory response, particularly those involved in the IL-1–mediated pathway, was in the range observed in the four patients with mild COVID-19. However, this upregulation was markedly lower than that observed in the patient with IRF9 deficiency, who also had mild COVID-19 (Fig. 5 A; Lévy et al., 2021). Thus, transcriptomic analysis demonstrated that the MyD88/IRAK-4–deficient patients were able to mount a systemic inflammatory response during the acute phase of the infection.

### Blood type I IFN transcriptome signature in patients during COVID-19

We collected whole-blood samples from four patients with IRAK-4 (P19, with moderate disease) or MyD88 (P1 with moderate disease, and P2 and P5 with severe disease) deficiency in the course of primary SARS-CoV-2 infection. These samples were used for whole-blood RNA-seq. Ex vivo transcriptome analysis showed that IRAK-4– and MyD88-deficient patients were able to produce type I IFNs during the acute phase of SARS-CoV-2 infection, as shown by the induction of IFN-stimulated genes (ISGs) in leukocytes, especially those with known antiviral functions (Fig. 5 B). The type I IFN activity detected in our patients was, as expected, much higher than that observed in an 8-yr-old girl with mild COVID-19 and an AR complete deficiency of IRF9 (Lévy et al., 2021), which governs ISGF-3–dependent responses to type I and III IFNs (Fig. 5 A). Indeed, a strong upregulation of numerous ISGs has been observed in peripheral blood during the first few days after symptom onset in patients displaying progression to severe disease relative to patients with mild disease (Galani et al., 2020; Hadjadj et al., 2020; Lee et al., 2020; Zhu et al., 2020; Kim and Shin, 2021; Ng et al., 2021; Ren et al., 2021; Zhao et al., 2021; Unterman et al., 2022). The scores obtained for our patients were in the range for control individuals with mild COVID-19 and higher than that obtained for a non-infected healthy control (Fig. 5 A). We also observed some heterogeneity in type I IFN activity between patients, possibly due to the timing of sampling after disease onset (with samples collected between 2 and 9 d after disease onset), or disease severity, which ranged from moderate and severe. These data suggest that despite the deficit of type I IFN production by pDCs, as in TLR7-deficient patients (Asano et al., 2021), other cells can produce type I IFNs that can activate leukocytes (Chiale et al., 2022; Lucas et al., 2020). However, the type I IFN signature was weaker than expected given the clinical severity of disease and relative to the general population infected with SARS-CoV-2 and analyzed in the first few days after symptoms onset.

### mAb-mediated neutralization of SARS-CoV-2 in an IRAK-4–deficient child

P19, a 14-yr-old male with IRAK4 deficiency, was admitted 2 d after the onset of clinical manifestation, including cough, nasal congestion, and fever (39.4°C). On day 1 of admission, PCR on a nasal swab revealed a very high load of SARS-CoV-2 (Ct Gene S: 16.20, Ct Gene N: 20.98, Ct Gene RdRP: 17.56), and showed the patient to be infected with the P681R/L425R Delta variant (previously known as B.1.617.2). A chest x-ray showed increased interstitial markings in the left retrocardiac space, but the SpO_2_/FIO_2_ ratio was 99%, indicating a moderate, non-hypoxemic, pneumonia. He then received a single dose of intravenous casirivimab (4,000 mg) and imdevimab (4,000 mg), a combination of human IgG1 neutralizing the receptor-binding domain of the SARS-CoV-2 spike protein on this same first day of admission. His symptoms and signs disappeared on day 3, and he was discharged on day 4. Follow-up evaluation on day 14 was unremarkable and the patient was asymptomatic.

We performed RNA-seq analysis on longitudinal whole-blood samples obtained from P19 at various time points from day 1 to 14. The transcripts of genes involved in antiviral immunity, particularly ISGs with known antiviral functions, were readily detected on days 1 and 2. Their levels decreased sharply on day 3, 4 d after symptom onset, when clinical manifestations disappeared, and, on day 4, they were barely detectable, if at all, as in a non-infected healthy control (Fig. 5 A). This pattern of expression contrasts with that observed in a patient with IRF9 deficiency and mild COVID-19 with positive PCR results for SARS-CoV-2, who was treated with casirivimab and imdevimab on day 2 after hospital admission (Lévy et al., 2021; Fig. 5 A). Moreover, the heatmap of RNA-seq–quantified gene expression (*z*-score–scaled log_2_-normalized counts) for TNF-α signaling through NF-κB gene sets showed lower transcript levels on admission in P19 than in the IRF9-deficient patient. Transcript levels for the induced genes of these inflammatory pathways decreased to very low levels 2 d after treatment with anti–SARS-Cov-2 mAbs in both patients (Fig. 5 B). These data, together with the rapid resolution of clinical manifestations in both patients, demonstrate the safety and efficacy of antibody-mediated viral neutralization in patients with either of these deficiencies of type I IFN–mediated immunity.

## Discussion

We showed that unvaccinated patients with MyD88 or IRAK-4 deficiency infected with SARS-CoV-2 are at high risk of COVID-19 pneumonia, including hypoxemic and even critical forms. The risk is much higher than for members of the same age group in the general population (Knock et al., 2021; Le Vu et al., 2021; Martin et al., 2022a; Martin et al., 2022b). The risk is also higher than that of heterozygous relatives, attesting to a lack of detectable co-dominance, and confirming that these two closely related IEIs confer a recessive predisposition to life-threatening COVID-19 pneumonia, even in childhood or adolescence. These patients appear to have a risk similar to that of children and adults with autoimmune polyendocrinopathy syndrome type 1 (APS-1), who are vulnerable to SARS-CoV-2 due to the production of auto-antibodies neutralizing type I IFNs (Bastard et al., 2021a; Lemarquis et al., 2021; Meisel et al., 2021). Our findings for patients with MyD88 or IRAK-4 deficiency are also consistent with previous findings from our own and other studies indicating that XR TLR7 deficiency confers a high risk of severe or critical COVID-19 pneumonia (van der Made et al., 2020; Asano et al., 2021; Fallerini et al., 2021; Kosmicki et al., 2021; Pessoa et al., 2021; Solanich et al., 2021a). The mechanism of disease in patients with MyD88 or IRAK-4 deficiency probably involves an impairment of the TLR7-mediated sensing of the virus by pDCs, as demonstrated ex vivo (Asano et al., 2021; Onodi et al., 2021)*.* The residual response of TLR7-deficient pDCs to SARS-CoV-2 ex vivo, contrasting with the abolition of this response in IRAK-4– and UNC-93B–deficient pDCs, may be due to signaling through TLR9, as pDCs do not express TLR8 (Aluri et al., 2021; Asano et al., 2021; Boisson and Casanova, 2021) and UNC-93B– and IRAK-4–deficient pDCs have defects of both TLR7 and TLR9 signaling (Onodi et al., 2021).

The TLR3-dependent induction of type I IFNs is intact in patients with MyD88 or IRAK-4 deficiency. Our findings, therefore, confirm that the TLR7-dependent induction of type I IFNs by pDCS is essential for host defense against SARS-CoV-2 in the respiratory tract. Patients with MyD88 or IRAK-4 deficiency seem to suffer from COVID-19 disease as severe as that in patients with TLR7 deficiency. Their more profound pDC defect, with the abolition of responses to both TLR7 and TLR9 agonists and a complete lack of type I IFN production upon stimulation with SARS-CoV-2 (Yang et al., 2005; Ku et al., 2007; von Bernuth et al., 2008; Alsina et al., 2014; Onodi et al., 2021), may be mitigated by other mechanisms, such as the abolition of responses to IL-1 paralogs, IL-18, and IL-33. The retrospective and prospective nature of the recruitment of TLR7- and MyD88/IRAK-4–deficient patients, respectively, may have led to an ascertainment bias. Nevertheless, the ISG response in our patients’ leukocytes was weak but detectable, probably due to activation of the TLR3-dependent or other pathways in infected lung epithelial cells. In addition, other sensors of viral RNA, such as RIG-1 and MDA-5, expressed in several leukocyte subsets, particularly in myeloid dendritic cells and monocytes, and at very low levels in resting pDCs, may have contributed to type I IFN production (Bencze et al., 2021; Liu and Gack, 2020). Noteworthy, transcriptome analyses have shown that the epithelial and immune cells of the upper airways of healthy children are preactivated and express significantly higher basal levels of the genes coding for RIG-I and MDA5 compared to adults, resulting in stronger innate antiviral responses upon SARS-CoV-2 infection (Loske et al., 2022). In addition, proteomic analyses, revealed that particularly RIG-I (also called DDX58) is among the most differentially detectable protein in circulation and in lung parenchymal tissues in patients with acute and fatal SARS-CoV-2 infection respectively vs. healthy controls (Filbin et al., 2021; Gisby et al., 2021; Russell et al., 2022). These data suggest that RIG-I may be a dominant pathway induced by the virus. On the other hand, there is a significant overlap between gene signatures for type I and type II IFN signaling. Our RNA-seq data do not allows to rule out that the IFN signature observed may be secondary, at least partially, to signaling by IFN-γ. Studies of the transcriptome of TLR3- or TLR7-deficient patients, and the identification of new IEIs underling hypoxemic COVID-19 pneumonia, would help to resolve these questions. Overall, impairment of the TLR7/MyD88/IRAK-4 pathway prevents pDCs from producing sufficient type I IFN in response to SARS-CoV-2 in the respiratory tract, accounting for the patients’ vulnerability to infections with this virus.

Since the first description of IRAK-4 and MyD88 deficiencies, the MyD88/IRAK-4–mediated pathway has been considered redundant for protective immunity against viruses in humans (Ku et al., 2007; Picard et al., 2010). EBV viremia without clinical repercussions was later reported in a MyD88-deficient patient (Chiriaco et al., 2019), and P5 was recently reported to have suffered from bilateral pneumonia caused by influenza A virus and the human cororonavirus NL63 (Bucciol et al., 2022a). An IRAK-4–deficient patient with a suspected reactivation of human herpesvirus 6 (HHV-6) infection has also been described (Nishimura et al., 2021), and genomic material from HHV-6 was also detected in three patients in our series (Table S2). A Turkish patient with severe HHV-6 disease has also recently been described (Tepe et al., 2022). The presence of the HHV-6 genome has, however, repeatedly been reported in normal brains, and HHV-6 reactivation may occur in healthy children without apparent illness or during acute illness (Caserta et al., 2004; Komaroff et al., 2020; Pandey et al., 2020; Santpere et al., 2020). Overall, the susceptibility of MyD88- and IRAK-4–deficient patients to HHV-6 is probable, but not formally proven. TLR7-deficient patients do not appear to be susceptible to common viral infections other than SARS-CoV-2, but further studies are required to confirm this. Indeed, TLR3 deficiency was initially reported in patients with herpes simplex encephalitis (Guo et al., 2011; Jouanguy et al., 2020; Zhang et al., 2007b), but patients with TLR3 deficiency were progressively found to be prone to other viral diseases too, including critical influenza pneumonia (Bucciol et al., 2022b; Kyung Lim et al., 2019), other types of viral encephalitis (Chen et al., 2021; Hautala et al., 2020; Kuo et al., 2022; Partanen et al., 2020), and hypoxemic COVID-19 pneumonia (Zhang et al., 2020b). Overall, our data suggest that MyD88 and IRAK-4 deficiencies underlie hypoxemic COVID-19 pneumonia. Together with the reports of HHV6 disease in such patients, and the occurrence of severe influenza pneumonia in two patients from our cohort, these data suggest that MyD88- and IRAK-4–deficient patients may be prone to other severe viral infections, perhaps with low penetrance.

## Materials and methods

### Cohort recruitment and consent

We recruited patients with MyD88 or IRAK-4 deficiency who had suffered COVID-19 between the start of the pandemic and January 2022. Data were collected through an anonymized survey sent to specialists in immunology or pediatrics with reported or unreported patients with these IEIs, and through clinicians caring for patients with IEIs identified from the European Society for Immunodeficiencies (ESID) Registry. Samples were obtained from the probands, parents and relatives with written informed consent. The study was approved by the French Ethics Committee “Comité de Protection des Personnes,” the French National Agency for Medicine and Health Product Safety, the “Institut National de la Santé et de la Recherche Médicale,” in Paris, France (protocol no. C10-13), the Rockefeller University Institutional Review Board in New York, NY, USA (protocol no. JCA-0700), the Committees for Ethical Research of the University Hospital of Gran Canaria Dr. Negrínn (protocol no. 2020-200-1 COVID-19) and Hospital San Joan de Deu (protocol no. PIC-173-21), and the Office of Research & Innovation at Drexel University, Philadelphia, PA, USA (protocol no. 2112008918).

### Definition of SARS-CoV-2 infection

SARS-CoV-2 infection was defined as a positive RT-PCR or antigenic test result for a nasopharyngeal sample for symptomatic patients, or as a positive serological test result for patients with no symptoms. The SARS-CoV-2 Ct value varied between patients. Viremia was not analyzed. The SARS-CoV-2 variants also differed between patients. In five patients, the viral variant was confirmed by molecular methods (two Alpha and three Delta variants). In the remaining patients, infection was suspected to be caused by the predominant variant in the country at the time of diagnosis (Our World in Data, 2023). 12 patients were infected between April 2020 and February 2021 when the variants of clades 20A and 20B, which replaced the original virus infecting humans (clade 19A), predominated. Four patients were infected in April–May 2021, when the variant of concern (VOC) Alpha (clade 20I, genetically confirmed in two) predominated. Three patients infected from November 2021 to January 2022 had genetically confirmed infections with the VOC Delta (clade 21A). Finally, three patients were infected in January 2022, when Omicron (clade 21M) was the predominant VOC in their countries of residence (Fig. 2 A and Table S1). For 15 patients, infection was confirmed to have resulted from household transmission from an infected relative (Table S1). In another two patients (P4 and P5), the infection was contracted from a visiting relative. The mode of infection of the remaining five patients is unknown. None of the patients were vaccinated against SARS-CoV-2 at the time of infection.

### Data regarding COVID-19 and medical history

Clinical, laboratory, and chest imaging data obtained during COVID-19, other risk factors for severe COVID-19 (Rodrigues et al., 2020; Zhang et al., 2020a, 2022a; Bennett et al., 2021; Bourgeois et al., 2021; Kooistra et al., 2021; Navaratnam et al., 2021; O’Driscoll et al., 2021; Westblade et al., 2021; Martin et al., 2022a; Schober et al., 2022; Woodruff et al., 2022), and family history were collected for each patient in the survey. Concomitant infections were also recorded, when supported by clinical suspicion, positive cultures, and/or chest x-ray images. Adults with a body mass index (BMI) over 25 were considered to be overweight, and those with a BMI over 30 were considered to be obese. Children aged between 5 and 19 yr were considered to be obese if their BMI-for-age-and-sex was more than 2 SD above the WHO Growth Reference median. Children under 5 yr of age were considered to be obese if their weight-for-height was more than 3 SD above the WHO Child Growth Standard median (WHO, 2021).

COVID-19 severity was assessed according to the Human Genetic Effort clinical score (Asano et al., 2021). SARS-CoV-2 infection was classified as mild/non-confirmed pneumonia (for patients who were asymptomatic, presented upper respiratory tract disease with no signs of pneumonia on x ray or with respiratory symptoms not suggestive of a lower respiratory tract infection and therefore not requiring x ray), moderate (non-hypoxemic pneumonia, not requiring oxygen therapy), severe (hypoxemic pneumonia requiring therapy with oxygen <6 liters O_2_/min, without meeting the criteria for critical pneumonia) or critical (hypoxemic pneumonia requiring high-flow oxygen >6 liters O_2_/min, ventilatory support with or without intubation, or ECMO [extracorporeal membrane oxygenation]).

Laboratory values were recorded when available. Normal ranges of laboratory values were reported according to age and are expressed in standard units (Hollowell et al., 2005; Mayo Clinic, 2022).

### Definition of positive contacts

Patients were considered to be positive SARS-CoV-2 contacts if they had been exposed (for more than 15 min, at a distance of <6 feet) to a person infected with the virus for whom an oral-nasopharyngeal sample had tested positive for specific SARS-CoV-2 RNA or antigen (CDC, 2022).

### Analysis of anti-type I IFN auto-Abs

The presence of auto-Abs able to neutralize high doses (10 ng/ml) of IFN-α2 and IFN-ω, and IFN-β, or lower, more physiological doses (100 pg/ml) of α2 and IFN-ω, was analyzed as previously reported (Bastard et al., 2021b) in plasma or serum samples from the patients.

### Next-generation sequencing

Genomic DNA was extracted from whole blood from all patients except P17, for whom DNA was obtained from SV40-transformed fibroblasts. The whole exome was sequenced at the Genomics Core Facility of the Imagine Institute (Paris, France), the Yale Center for Genome Analysis the New York Genome Center, and The American Genome Center (Uniformed Services University of the Health Sciences, Bethesda, MD, USA), and the Genomics Division–Institute of Technology and Renewable Energies of the Canarian Health System sequencing hub (Canary Islands, Spain), as previously reported (Asano et al., 2021). The whole-exome sequences of the patients were filtered against the complete International Union of Immunological Societies list of genes (Tangye et al., 2022), with the retention of variants with an allele frequency below 0.001. We excluded synonymous mutations, downstream, upstream, intron and non-coding transcript variants and intergenic variants. We also excluded variants predicted to be benign and we checked the quality of the exome sequences. The mutation significance cutoff (http://pec630.rockefeller.edu:8080/MSC/) was used to determine whether variants were likely to be damaging.

### RNA-seq

Whole blood for RNA-seq was collected in PAXgene Blood RNA tubes (BD Biosciences, samples collected from the IRF9-deficient patient, her mother, P5, and P19), Tempus Blood RNA tubes (Thermo Fisher Scientific, P1 and P2) or EDTA tubes (controls). Samples from P1 (moderate pneumonia) were obtained at hospital admission, 8 d after positive PCR and 2 d after symptom onset; samples from P2 (severe pneumonia) were collected on hospital admission, 4 d after PCR and symptom onset; samples from P5 (severe pneumonia) were obtained 6 d after hospital admission, 9 d after PCR and symptom onset; and the first sample from P19 was obtained before treatment with anti–SARS-CoV-2 mAbs, on the day of hospital admission, 2 d after symptom onset, when PCR was performed. We also collected longitudinal whole-blood samples from P19 at various time points from day 1 to 14. We compared our data with data obtained for samples from four controls with mild COVID-19 obtained 4 d after a positive PCR for SARS-CoV-2, a non-infected healthy control, and the longitudinal data (day 1 [hospitalization] to day 32), obtained from a previously reported IRF9-deficient patient with mild SARS-CoV-2 infection also treated with anti–SARS-CoV-2 mAbs (Lévy et al., 2021) the day after hospital admission.

There was one sample for each time-point and patient and the assay was performed once for each sample.

Blood samples were subjected to hemoglobin RNA depletion. Samples were sequenced on the Illumina NextSeq platform with a single-end 75-bp configuration. The RNA-seq fastq raw data were inspected to ensure that they were of high quality. The sequencing reads were mapped onto the human reference genome GRCh38 with STAR aligner v.2.7, and the mapped reads were then quantified with featureCounts v2.0.2 to determine gene-level read counts. The gene-level read counts were normalized and log_2_-transformed with DESeq2, to obtain the gene expression profile for all samples.

Single-sample gene set enrichment analysis (Hänzelmann et al., 2013) was used to evaluate the IFN-α response, IFN-γ response, TNF-α signaling through NF-κB, IL-6 JAK-STAT3 signaling, including the inflammatory response enrichment scores. The raw RNA-seq data generated from this study are deposited in the National Center for Biotechnology Information database under the National Center for Biotechnology Information- Sequence Read Archive project PRJNA916275.

### Statistical analysis

Categorical variables are expressed as percentages, and discrete variables as medians with the observed range, or as means ±95% CI. Fisher’s exact tests or Yates correction and ORs with 95% CI were used for comparative analyses. Continuous variables are presented as the arithmetic mean ± SD, and Mann–Whitney U*-*tests were used for the comparative analysis. The analysis was performed with SPSS v.15.0 software (SPSS, Inc.) and graphs were performed by GraphPad Prism v.7.00 for Windows, GraphPad Software, with values of P ≤ 0.05 considered statistically significant.

### Online supplemental material

Table S1 provides information about baseline characteristics of MyD88 and IRAK-4–deficient patients, data on the diagnosis of COVID-19 and the lung conditions during the infection. Table S2 contains information about non–SARS-CoV-2 viral infections in the MyD88 and IRAK-4–deficient patients. Table S3 shows the serological results for antibodies against common viruses for two patients. Table S4 summarizes the pLOF variants of the 478 genes known to underlie AR, AD, or XR IEIs in our MyD88/IRAK-4–deficient patients. Table S5 includes the laboratory data of MyD88 and IRAK-4–deficient patients during SARS-CoV-2 infection.

## Supplementary Material

Table S1 shows baseline medical characteristics of MyD88 and IRAK-4–deficient patients, diagnosis of COVID-19, and lung conditions during SARS-CoV-2 infection.

Table S2 shows other viral infections documented in our patients.

Table S3 shows serological results for antibodies against common viruses for two patients.

Table S4 shows rare pLOF variants of the 478 genes known to underlie AR, AD, or XR IEIs in patients with MyD88/IRAK-4 deficiency with SARS-CoV-2 infection.

Table S5 shows laboratory data of MyD88 and IRAK-4–deficient patients during SARS-CoV-2 infection.
