# Supplementary material for: Humans with inherited MyD88 and IRAK-4 deficiencies are predisposed to hypoxemic COVID-19 pneumonia
Source: J Exp Med. 2023 Mar 3;220(5):e20220170. doi: 10.1084/jem.20220170 (PMC9998661; doi:10.1084/jem.20220170)
Supplement: Table S1 — shows baseline medical characteristics of MyD88 and IRAK-4–deficient patients, diagnosis of COVID-19, and lung conditions during SARS-CoV-2 infection. [file JEM_20220170_TableS1.docx]

**Table S1.** Baseline medical characteristics of MyD88 and IRAK-4–deficient patients, diagnosis of COVID-19, and lung conditions during SARS-CoV-2 infection

| **Kindred/**  **Patient** | **Age (yr) and gender** | **IEI** | **IEI baseline treatment** | **SARS-CoV-2 diagnostic method/PCR-Ct^a^** | **SARS-CoV-2–positive serologies^b^** | **SARS-CoV-2 variant^c^** | **Days since positive contact (BA)^d^** | **Days since first symptoms (BA)^e^** | **Positive household relatives^f^** | **Min spO2 (%)** | **Suspected bacterial superinfection** | **Previous reports** |
| --- | --- | --- | --- | --- | --- | --- | --- | --- | --- | --- | --- | --- |
| **A/P1** | 17 (F) | MyD88 | No | NP Ag/NA | IgG, IgM (12) | (20A, original) | 8 | 2 | Yes (father *C*, mother, P2 wife) | >95 | Yes  Chest x ray: pleural effusion. | Von Bernuth et al., 2008; Deyà-Martínez et al., 2021 |
| **A/P2** | 19 (M) | MyD88 | No | NP PCR/NA | IgG, IgM (11) | (20A, original) | 15 | 4 |  | 85 | No |  |
| **B/P3** | 6 (M) | MyD88 | Amoxicillin  IgRT | NP PCR/ 26.86(E) | IgG (30) | (20B, original) | Unk. | Unk. | Yes (father, mother *C*) | >95 | Unk. (no chest X-ray) | No |
| **C/P4** | 1.5 (M) | MyD88 | Amoxicillin, cotrimoxazole  IgRT | NP PCR/NA | IgG, IgM (14) | (20I, alpha) | 1 | 1 | Yes (father, mother, brother; infection was contracted from a visiting relative) | >95 | No  *P.aeruginosa* in pharingeal swab, no compatible X-ray. | No |
| **D/P5** | 6.5 (M) | MyD88 | Amoxicillin  IgRT | NP PCR/ 11.07(E) | ND | (20I, alpha) | 7 | 3 | Yes (father and mother, infection was contracted from a visiting relative) | 88 | Yes  Improvement upon antibiotics.  Chest X-ray: lung consolidations. | Bucciol el al., 2022b |
| **E/P6** | 16 (F) | MyD88 | IgRT | NP PCR/ 8.6(S) | IgG (ND) | (20A/20B, original) | 5 | 3 | Yes (father *C*) | 85 | Yes  Chest X-Ray: multifocal pneumonia.  Respiratory cultures: negative. | Conway et al., 2010 |
| **E/P7** | 15 (F) | MyD88 | IgRT | NP PCR/ 8.9(S) | IgG (23) | (20A/20B, original) | 5 | 3 |  | 80 | Yes  Chest X-ray: pleural effusion, lung consolidations.  Respiratory cultures: negative |  |
| **E/P8** | 12 (M) | MyD88 | IgRT | NP PCR/  19.4(S) | ND | (20A/20B, original) | 5 | 3 |  | >95 | No |  |
| **F/P9** | 14 (M) | MyD88 | IgRT | NP PCR/NA | ND | (20A/20B, original) | 16 | 5 | Yes (father, mother *C*) | 88 | No | Conway et al., 2010 |
| **F/P10** | 9 (M) | MyD88 | IgRT | NP PCR/NA | ND | (20A/20B, original) | 16 | 1 |  | 90 | Yes  X-ray: lobar pneumonia | No |
| **G/P11** | 8 (M) | MyD88 | Cotrimoxazole  IgRT | Serology/NA | ND | (20I, alpha) | Unk. | NA | Unk. | >95 | Unk. (no chest X-ray) | Milito et al., 2021 |
| **H/P12** | 8 (M) | MyD88 | Amoxicillin, cotrimoxazole  IgRT | NP PCR/ <25(ND) | ND | 20I, alpha | 3 | 2 | Yes (father *C*) | 93 | Yes  Chest X-ray: lobar pneumonia | No |
| **I/P13** | 13 (M) | MyD88 | Amoxicillin, cotrimoxazole | NP PCR/NA | ND | (20B, original) | Unk. | 1 | Unk. | >95 | No  NP swab: rhinovirus | No |
| **J/P14** | 1.2 (M) | MyD88 | No | NP PCR/ 23(S) | ND | 21A, delta | Unk. | 7 | Yes (father, mother, grandmother, no *C* identified) | 60 | Yes  Chest X-ray: bilateral consolidations. | No |
| **K/P15** | 0.17 (M) | MyD88 | No | NP PCR/ <30(ND) | ND | 21A, delta | NA | 3 | Yes (mother *C*, father) | 70 | No  X-ray: diffuse infiltrates | No |
| **I/P16** | 24 (M) | IRAK4 | No | NP PCR/  21.83(E) | IgG (4) | 20I, alpha | 7 | 6 | Yes (father, mother *C*, sister and brother) | >95 | Yes  Pneumococcal antigen in urine, (chest X-ray: compatible with COVID-19 pneumonia) | Yang et al., 2005; Cardenes et al., 2006; von Bernuth et al., 2006; Ku et al., 2007; Weller et al., 2012; Picard et al., 2010 |
| **M/P17** | 23 (M) | IRAK4 | Amoxicillin  IgRT | NP PCR/NA | IgG (ND) | (20A/20B, original) | Unk. | 3 | Yes (mother, no *C* identified) | Death  (at hospital admission SpO_2_/FIO_2_ 82%) | Yes  Respiratory cultures: MRSA, Citrobacter Chest X-ray: consolidations, cavitation and pleural effusion. | Picard et al., 2010 |
| **N/P18** | 15 (M) | IRAK4 | No | NP PCR/NA | ND | (20A, original) | 5 | 5 | Yes (26 years-old brother, no *C* identified) | >95 | No | No |
| **O/P19** | 14 (M) | IRAK4 | Cotrimoxazole  IgRT | NP PCR/ 16.20(S) | IgG (15) | 21A, delta | 7 | 2 | Yes (mother *C*, 2 sisters) | >95 | No | No |
| **P/P20** | 6 (M) | IRAK4 | Amoxicillin  IgRT | NP Ag/NA | ND | (21M, omicron) | NA | NA | Yes (mother *C*, fahter) | ND | Unk. (no chest X-ray) | Jia et al., 2020 |
| **P/P21** | 3 (M) | IRAK4 | Amoxicillin  IgRT | NP Ag/NA | ND | (21M, omicron) | NA | NA |  | ND | Unk. (no chest X-ray) |  |
| **Q/P22** | 8 (F) | IRAK4 | Cotrimoxazole  IgRT | NP PCR/NA | IgG (70) | (21M, omicron) | NA | NA | Yes (mother and brother *C*, father, 2 brothers) | ND | Unk. (no chest X-ray) | No |

# P, patient; M: male, F, female; proph, prophylactic; IgRT, immunoglobulin replacement therapy; NP, nasopharyngeal; Ag, antigen; BA, before admission; SpO2, oxygen saturation measured by fingertip pulse oximeter; MRSA, Methicillin-resistant *S.aureus*; Unk., unknown; ND, no data; *C*, positive contact.

## ^a^Technique used for the infection diagnosis/Ct (gen used)

## ^b^Specific positive serologies to SARS-CoV-2 (IgG and IgM) during the acute infection (days after the infection was diagnosed)

## ^c^SARS-CoV-2 variant: parenthesis are used for those variants that were not analyzed in the patient but inferred from the most prevalent variant in the area and period of time (<https://ourworldindata.org/grapher/covid-variants-area>); the variants of the clades 20A and 20B replaced the considered original virus infecting humans (clade 19A), data about the variants of concern alpha (clade 20I), delta (clade 21A), and omicron (clade 21M) are also shown.

## ^d^Days from the contact to the positive SARS-CoV-2 carrier to the hospital admission, or if the contact is unknown

## ^e^Days since the first symptoms appeared until the patient was admitted in the hospital

## ^f^Patient sharing home with positive relatives: Yes, No, Unk., (positive relatives, *C* if they were the first known positive contact).

**References**

Bucciol, G., L. Moens, A. Corveleyn, A. Dreesman, and I. Meyts. 2022b. A novel kindred with MyD88 deficiency. *J. Clin. Immunol.* 42:885–888. 10.1007/s10875-022-01240-6

Cardenes, M., H. von Bernuth, A. García-Saavedra, E. Santiago, A. Puel, C.L. Ku, J.F. Emile, C. Picard, J.L. Casanova, E. Colino, et al. 2006. Autosomal recessive interleukin-1 receptor-associated kinase 4 deficiency in fourth-degree relatives. *J. Pediatr.* 148:549–551. 10.1016/j.jpeds.2005.12.012

Conway, D.H., J. Dara, A. Bagashev, and K.E. Sullivan. 2010. Myeloid differentiation primary response gene 88 (MyD88) deficiency in a large kindred. *J. Allergy Clin. Immunol.* 126:172–175. 10.1016/j.jaci.2010.04.014

Deyà-Martínez, A., A. García-García, E.A. Gonzalez-Navarro, L. Yiyi, A. Vlagea, I. Jordan, V. Fumadó, C. Fortuny, M. Español, C. Launes, et al. 2021. COVID-19 in children and young adults with moderate/severe inborn errors of immunity in a high burden area in pre-vaccine era. *Clin. Immunol.* 230:108821. 10.1016/j.clim.2021.108821

Jia, A., E. James, H.Y. Lu, M. Sharma, B.P. Modi, C.M. Biggs, K.J. Hildebrand, A. Chomyn, S. Erdle, H. Kular, and S.E. Turvey. 2020. Clinical IRAK4 deficiency caused by homozygosity for the novel IRAK4 (c.1049delG, p.Gly350Glufs*15) variant. *Cold Spring Harb. Mol. Case Stud*. 6:a005298. 10.1101/mcs.a005298

Ku, C.L., H. von Bernuth, C. Picard, S.Y. Zhang, H.H. Chang, K. Yang, M. Chrabieh, A.C. Issekutz, C.K. Cunningham, J. Gallin, et al. 2007. Selective predisposition to bacterial infections in IRAK-4-deficient children: IRAK-4-dependent TLRs are otherwise redundant in protective immunity. *J. Exp. Med.* 204:2407–2422. 10.1084/jem.20070628

Milito, C., V. Lougaris, G. Giardino, A. Punziano, A. Vultaggio, M. Carrabba, F. Cinetto, R. Scarpa, R.M. Delle Piane, L. Baselli, et al. 2021. Clinical outcome, incidence, and SARS-CoV-2 infection-fatality rates in Italian patients with inborn errors of immunity. *J. Allergy Clin. Immunol. Pract.* 9:2904–2906.e2. 10.1016/j.jaip.2021.04.017

Picard, C., H. von Bernuth, P. Ghandil, M. Chrabieh, O. Levy, P.D. Arkwright, D. McDonald, R.S. Geha, H. Takada, J.C. Krause, et al. 2010. Clinical features and outcome of patients with IRAK-4 and MyD88 deficiency. *Medicine.* 89:403–425. 10.1097/MD.0b013e3181fd8ec3

von Bernuth, H., C.L. Ku, C. Rodriguez-Gallego, S. Zhang, B.Z. Garty, L. Maródi, H. Chapel, M. Chrabieh, R.L. Miller, C. Picard, et al. 2006. A fast procedure for the detection of defects in Toll-like receptor signaling. *Pediatrics*. 118:2498–2503. 10.1542/peds.2006-1845

von Bernuth, H., C. Picard, Z. Jin, R. Pankla, H. Xiao, C.L. Ku, M. Chrabieh, I.B. Mustapha, P. Ghandil, Y. Camcioglu, et al. 2008. Pyogenic bacterial infections in humans with MyD88 deficiency. *Science*. 321:691–696. 10.1126/science.1158298

Weller, S., M. Bonnet, H. Delagreverie, L. Israel, M. Chrabieh, L. Maródi, C. Rodriguez-Gallego, B.Z. Garty, C. Roifman, A.C. Issekutz, et al. 2012. IgM+IgD+CD27+ B cells are markedly reduced in IRAK-4-, MyD88-, and TIRAP- but not UNC-93B-deficient patients. *Blood.* 120:4992–5001. 10.1182/blood-2012-07-440776

Yang, K., A. Puel, S. Zhang, C. Eidenschenk, C.L. Ku, A. Casrouge, C. Picard, H. von Bernuth, B. Senechal, S. Plancoulaine, et al. 2005. Human TLR-7-, -8-, and -9-mediated induction of IFN-α/β and -λ Is IRAK-4 dependent and redundant for protective immunity to viruses. *Immunity.* 23:465–478. 10.1016/j.immuni.2005.09.016
