# Supplementary material for: Humans with inherited MyD88 and IRAK-4 deficiencies are predisposed to hypoxemic COVID-19 pneumonia
Source: J Exp Med. 2023 Mar 3;220(5):e20220170. doi: 10.1084/jem.20220170 (PMC9998661; doi:10.1084/jem.20220170)
Supplement: Table S2 — shows other viral infections documented in our patients. [file JEM_20220170_TableS2.docx]

**Table S2.** Other viral infections documented in our patients

| **Patient** | **Previous viral infections (age)** | **COVID-19 severity**  **(age; yr)** |
| --- | --- | --- |
| **P1** | EBV viremia (max 164790 copies/ml), IgM positive serology, without clinical repercussion (4 yr).  Positive cutaneous HSV-1 (PCR) without systemic symptoms (7 yr) | Moderate (17) |
| **P2** | Mild H1N1pdm09 infection (8 yr) | Severe (19) |
| **P5** | Severe influenza A virus and coronavirus NL63 bilateral pneumonia (3 yr)  Recurrent pharyngitis | Severe (6.5) |
| **P11** | Encephalitis by *S. pneumoniae* and positive PCR for HHV-6 (7.5 yr) | Asymptomatic (8) |
| **P12** | Myopericarditis (9 months) (no viral etiology confirmed) | Moderate (8) |
| **P14** | High HBV viremia, 3,451 millions copies/ml) without clinical manifestations (HBs and HBe antigens positive, anti-HBs and anti-HBc negative; 1 yr)  Encephalitis by *S. agalactiae* and positive PCR for HHV-6 (8 mo) | Critical (1) |
| **P16** | Mild bronchospasm associated to H1N1pdm09 influenza infection (12 yr) | Moderate (24) |
| **P17** | Mild RSV (8 mo)  Mild hMPV (17 mo)  Mild Influenza B virus (4 yr and 9 mo)  Severe H1N1pdm09 pneumonia later complicated with a secondary bacterial pneumonia (13 yr). | Critical (23) |
| **P19** | EBV infection without clinical repercussion (5 yr) | Mild (14) |
| **P22** | Cutaneous VZV infection with fever during 2 d and no systemic complications, treated with acyclovir (2 yr and 6 mo)  PCR positive in blood samples for HHV-6 when he developed direct Coombs-positive anemia and was on rapamycin and corticosteroids maintenance treatment for auto-immune hepatitis (3 yr and 8 mo) | Mild/non-confirmed pneumonia (8) |

H1N1pdm09, pandemic H1N1 influenza virus 2009; HHV-6, human herpesvirus 6 (not data about HHV-6A or HHV-6B was documented); HBV, hepatitis B virus; RSV, respiratory syncytial virus; hMPV, human metapneumovirus; VZV, varicella-zoster virus.
