# Supplementary material for: Humans with inherited MyD88 and IRAK-4 deficiencies are predisposed to hypoxemic COVID-19 pneumonia
Source: J Exp Med. 2023 Mar 3;220(5):e20220170. doi: 10.1084/jem.20220170 (PMC9998661; doi:10.1084/jem.20220170)
Supplement: Table S3 — shows serological results for antibodies against common viruses for two patients. [file JEM_20220170_TableS3.docx]

**Table S3.** Serological results for antibodies against common viruses for two patients

| **Parameters** | **P13^a^**  **(13 yr)** | **P18^b^**  **(15 yr)** | **Threshold** |
| --- | --- | --- | --- |
| IgG anti-HSV-1 | 32 | 55.1 | >1.1 |
| IgG anti-HSV-2 | <0.5 | <0.5 | >1.1 |
| IgG anti-VZV | 45.2 | 1,653 | >135 |
| IgG anti-EBNA (EBV) | 9.64 | 460 | >20 |
| IgG anti-VCA (EBV) | 221 | 195 | >20 |
| IgG anti-CMV | <5 | 101 | >14 |
| IgG anti-HAV | 0.42 | 9.52 | >1 |
| IgG anti-parvovirus B19 | 0.10 | 6 | >1.1 |
| IgG anti-measles | 82^a^ | 268^b^ | >16.5 |
| IgG anti-mumps | 259^a^ | 39,5 | >11 |
| IgG anti-rubella | 67.1^a^ | 67.8^b^ | >11 |

HSV-1, herpes simplex virus 1; HSV-2, herpes simplex virus 2; VZV, varicella-zoster virus; EBNA, Epstein–Barr nuclear antigen; VCA, viral-capsid antigen; HAV: hepatitis A virus.

^a^P13 was previously vaccinated with anti-Measles/Mumps/Rubella vaccine.

^b^P18 was previously vaccinated with anti-Measles/Rubella vaccine.
