# Supplementary material for: Humans with inherited MyD88 and IRAK-4 deficiencies are predisposed to hypoxemic COVID-19 pneumonia
Source: J Exp Med. 2023 Mar 3;220(5):e20220170. doi: 10.1084/jem.20220170 (PMC9998661; doi:10.1084/jem.20220170)
Supplement: Table S4 — shows rare pLOF variants of the 478 genes known to underlie AR, AD, or XR IEIs in patients with MyD88/IRAK-4 deficiency with SARS-CoV-2 infection. [file JEM_20220170_TableS4.docx]

**Table S4.** Rare pLOF variants of the 478 genes known to underlie AR, AD, or XR IEIs in patients with MyD88/IRAK-4 deficiency with SARS-CoV-2 infection

| **Patient** | **COVID-19 severity** | **Gene** | **Chr** | **Position (GRCh37)** | **Reference** | **Altered** | **Variant** | **Zygosity** | **Inheritance^a^** | **CADD (MSC)** | **ID** | **AF** |
| --- | --- | --- | --- | --- | --- | --- | --- | --- | --- | --- | --- | --- |
| **P1** | Moderate | *NBAS* | 2 | 15644308 | C | T | p.M305I | Het | AR | 9.744 (3.313) | rs562939436 | 0.000322 |
| **P2** | Severe | *NBAS* | 2 | 15644308 | C | T | p.M305I | Het | AR | 9.744 (3.313) | rs562939436 | 0.000322 |
|  |  | *CSF2RB* | 22 | 37331458 | C | T | p.R461C | Het | AR | 24.800 (24.700) | rs371045078 | 0.0002029 |
| **P3** | Mild/NCP | *POL3RA* | 10 | 79742529 | C | T | p.R1159H | Het | AD | 24.900 (3.102) | rs150484386 | 0.0002068 |
| **P4** | Mild/NCP | *TOP2B* | 3 | 25661382 | G | C | p.L1007V | Het | AD | 25.300 (5.442) | - | - |
|  |  | *VPS13B* | 8 | 100874122 | C | G | p.S3746R | Het | AR | 27.700 (0.002) | - | - |
|  |  | *CLCN7* | 16 | 1515269 | G | A | p.P71L | Het | AR | 22.500 (4.477) | rs145267254 | 0.0002796 |
| **P5** | Severe | *--NM--*^b^ |  |  |  |  |  |  |  |  | - | - |
| **P13** | Moderate | TBX21 | 17 | 45820443 | G | A | p.R218H | Het | AR | 31.000 (3.313), | rs202150196 | 0.0001275 |
| **P14** | Critical | *TET2* | 4 | 106162582 | G | A | p.E1166K | Het | AR | 24.900 (0.386) | - | - |
|  |  | *RTEL1* | 20 | 62324579 | C | T | p.R756W | Het | AD | 22.000 (10.050) | rs144034326 | \| 0.0001084 \|  \| \| --- \| --- \| |
|  |  | ADA2 | 22 | 17663560 | G | C | p.H150Q | Het | AR | 24.100 (6.827) | rs780182069 | \| 0.00001988 \|  \| \| --- \| --- \| |
| **P15** | Critical | *IFNAR1* | 21 | 34713344 | G | C | p.Q80H | Het | AR, AD (COVID-19) | 11.690 (3.313) | rs1333470928 | \| 0.000004 \|  \| \| --- \| --- \| |
|  |  | *POL3RA* | 10 | 79742529 | C | T | p.R1159H | Het | AD | 24.900 (3.102) | rs150484386 | 0.0002068 |
|  |  | *FERMT1* | 20 | 6068525 | C | T | p.E424K | Het | AR | 35.000 (0.025) | rs747141428 | 0. 0000956 |
| **P16** | Moderate | *LIG1* | 19 | 48626566 | G | A | p.R604C | Het | AR | 35.000 (33.000) | rs55950593 | 0.0001837 |
|  |  | *NBAS* | 2 | 15378623 | C | G | p.G5912C | Het | AR | 22.000 (3.313) | rs754178038 | 0.000004 |
|  |  | *G6PC3* | 17 | 42152722 | C | T | p.A154V | Het | AR | 29.200 (11.340) | rs748638373 | 0.000008 |
| **P17** | Critical | *PMS2* | 7 | 6022617 | G | A | p.T671M | Het | AR | 27.800 (0.025) | rs587780046 | 0.0002791 |
|  |  | *APOL1* | \| 22 \| 36651020 \| \| --- \| --- \| | 36651020 | C | G | p.L23V | Homo | AD | 18.910 (3.312) | rs372661836 | 0.00004375 |
| **P18** | Moderate | *RTEL1* | 20 | 62324326 | G | A | p.E941K | Het | AD | 21.700 (10.050) | rs906116592 | 0.00003187 |
| **P19** | Moderate | *UNC13D* | 17 | 73840387 | C | G | p.R11P | Het | AR | 29.600 (0.001) | rs574927621 | 0.00008476 |
|  |  | *FOXN1* | 17 | 26851767 | C | A | p.H124N | Het | AR | 26.400 (24.900) | - | - |
|  |  | *VPS13B* | 8 | 100533180 | A | G | p.M158V | Het | AR | 9.086 (0.002) | rs886062541 | - |
|  |  | *TFRC* | 3 | 195798995 | G | A | p.R155C | Het | AR | 34.000 (3.313) | rs377519674 | 0.00001992 |
|  |  | *FAT4* | 4 | 126239839 | C | T | p.A758V | Het | AR | 14.370 (0.001) | rs570502199 | 0.00009226 |
| **P20** | Mild/NCP | *FANCD2* | 3 | 10106408 | C | T | p.F386V | Het | AR | 10.640 (0.001) | rs4019784 | 0.0002771 |
|  |  | *C7* | 5 | 40937657 | C | A | p.Y144X | Het | AR | 36.000 (0.001) | rs780119460 | 0.000618 |
|  |  | *CARD9* | 9 | 139265456 | C | T | p.R155H | Het | AR | 22.300 (0.699) | rs145198648 | 0.00009798 |
|  |  | *OAS1* | 12 | 113355369 | C | T | p.P301L | Het | AD GOF | 31.000 (3.313) | rs201971047 | 0.00007161 |
| **P21** | Mild/NCP | *IGLL1* | 22 | 23915703 | G | A | p.A131V | Het | AR | 24.000 (3.313) | rs766919157 | 0.00001593 |
|  |  | *FANCD2* | 3 | 10088285 | T | G | p.F386V | Het | AR | 10.370 (0.001) | rs149125003 | 0.00004397 |
|  |  | *OAS1* | 12 | 113355369 | C | T | p.P301L | Het | AD GOF | 31.000 (3.313) | rs201971047 | 0.00007161 |
|  |  | *FANCM* | 14 | 45658564 | A | AT | p.K1780fs | Het | AR | 35.000 (34.000) | rs752637629 | 0.00006379 |
|  |  | *C7* | 5 | 40937657 | C | A | p.Y144X | Het | AR | 36.000 (0.001) | rs780119460 | 0.000618 |
| **P22** | Mild/NCP | *CFTR* | 7 | 117149146 | C | T | p.R75X | Het | AR | 37.000 (0.001) | rs121908749 | \| 0.00002391 \|  \| \| --- \| --- \| |

ID, identifier; AF, allele frequency; Anti-IFNs-I autoAb, anti-type I IFNs auto-Abs able to neutralize IFN-α and/or -ω and/or -β; Het, heterozygous; Hom, homozygous; GOF, gain of function; NCP, non-confirmed pneumonia.

^a^Inheritance of inborn errors of immunity (Tangye et al., 2022).

^b^--NM--: No candidate mutations were found.

References

Tangye, S.G., W. Al-Herz, A. Bousfiha, C. Cunningham-Rundles, J.L. Franco, S.M. Holland, C. Klein, T. Morio, E. Oksenhendler, C. Picard, et al. 2022. Human inborn errors of immunity: 2022 update on the classification from the international union of immunological societies expert committee. *J. Clin. Immunol.* 42:1473–1507. 10.1007/s10875-022-01289-3
