# Supplementary material for: Humans with inherited MyD88 and IRAK-4 deficiencies are predisposed to hypoxemic COVID-19 pneumonia
Source: J Exp Med. 2023 Mar 3;220(5):e20220170. doi: 10.1084/jem.20220170 (PMC9998661; doi:10.1084/jem.20220170)
Supplement: Table S5 — shows laboratory data of MyD88 and IRAK-4–deficient patients during SARS-CoV-2 infection. [file JEM_20220170_TableS5.docx]

**Table S5.** Laboratory data of MyD88 and IRAK-4–deficient patients during SARS-CoV-2 infection

|  | **Lymphocytes (cells/μl)** | **NR** | **Neutrophils (cells/μL)** | **NR** | **Platelets (10^3^cells/μl)** | **NR** | **PCT (ng/ml)** | **NR** | **CRP (mg/liter)** | **NR** | **Ferritin (ng/ml)** | **NR** | **D-dimer (μg/l)** | **NR** | **LDH (IU/liter)** | **NR** | **Bilirubin (mg/dl)** | **NR** | **Creatinine (mg/dl)** | **NR** |
| --- | --- | --- | --- | --- | --- | --- | --- | --- | --- | --- | --- | --- | --- | --- | --- | --- | --- | --- | --- | --- |
| **P1** | 600/3100 | 1250-3650 | 5800/4200/5800 | 1900-9310 | 167/303 | 158-362 | 0.1/0.1 | ≤0.15 | 78.3/108.6 | ≤8.0 | 118.4/310.6 | 10-120 | 1940/1970 | ≤500 | 824/824 | 105-233 | 0.4 | ≤1.0 | 0.61 | 0.59-1.04 |
| **P2** | 900/1900 | 1250-3650 | 5600/5600/6700 | 1900-9310 | 242/413 | 135-317 | 0.25/ND | ≤0.15 | 87.4/ND | ≤8.0 | 305.8/ND | 20-250 | 410/ND | ≤500 | 834/879 | 122-222 | ND | - | 0.98 | 0.74-1.35 |
| **P3** | 3370/ND | 1500-4800 | 4130/ND/ND | 1750-8190 | 283/ND | 187-400 | 0.04/ND | ≤0.15 | 0.2/ND | ≤8.0 | ND | - | ND | - | 350/ND | 145-345 | ND | - | 0.58 | 0.26-0.61 |
| **P4** | 3520/ND | 1500-5500 | 2030/ND/ND | 1200-7590 | 316/ND | 206-445 | 0.08/ND | ≤0.15 | 6.5/ND | ≤8.0 | 50 | 7-140 | ND | - | ND | - | 0.16 | ≤1.0 | 0.49 | 0.19-0.49 |
| **P5** | 1000/2570 | 1500-4800 | 1520/1520/4005 | 1750-8190 | 265/ND | 187-400 | ND | - | 52/59 | ≤8.0 | ND | - | ND/631 | ≤500 | 408/553 | 145-345 | ND | - | 0.47 | 0.26-0.61 |
| **P6** | 900/3300 | 1250-3650 | 5300/520/5300 | 1900-9310 | 136/377 | 158-362 | 1.43/1.43 | ≤0.15 | 106.7/197.5 | ≤8.0 | 448/1440 | 10-120 | 193/300 | ≤500 | 335/513 | 105-233 | 1.37 | ≤1.2 | 0.78 | 0.56-1.04 |
| **P7** | 1200/5900 | 1250-3650 | 4900/900/5500 | 1900-9310 | 168/241 | 158-362 | 7.26/7.26 | ≤0.15 | 117.3/173.3 | ≤8.0 | 556/3347 | 10-120 | 304/2000 | ≤500 | 518/1028 | 110-283 | 1.81 | ≤1.2 | 0.61 | 0.35-0.86 |
| **P8** | 1600/3000 | 1450-4450 | 5400/900/6000 | 1700-8050 | 192/296 | 177-381 | 0.11/0.11 | ≤0.15 | 14.34/126.1 | ≤8.0 | 240/253 | 7-140 | 51/100 | ≤500 | 251/252 | 110-283 | ND | - | 0.57 | 0.35-0.86 |
| **P9** | 700/1600 | 1450-4450 | 3900/2500/4500 | 1700-8050 | 158/307 | 139-320 | ND | - | 187.0/187.0 | ≤8.0 | ND | 7-140 | 110/100 | ≤500 | ND | - | 0.81 | ≤1.2 | 0.58 | 0.35-0.86 |
| **P10** | 1300/3300 | 1400-4350 | 2300/2300/10700 | 1600-8200 | 251/408 | 187-400 | ND | - | 33.3/33.3 | ≤8.0 | ND | 7-140 | 105/100 | ≤500 | ND | - | 0.71 | ≤1.2 | 0.35 | 0.26-0.61 |
| **P11** | ND | - | ND | - | ND | - | ND | - | ND | - | ND | - | ND | - | ND | - | ND | - | ND | - |
| **P12** | 1200/4725 | 1500-4800 | 2900/ND/ND | 1750-8190 | 197/ND | 187-400 | unk./0.9 | ≤0.15 | 20.4/20.4 | ≤8.0 | ND | - | ND | - | 382/382 | 143-290 | ND/0.7 | ≤1.2 | 0.38/0.38 | 0.26-0.61 |
| **P13** | 700/7000 | 1450-4450 | 2260/1700/2260 | 1700-8050 | 193/ND | 177-381 | ND | - | 43.9/69.1 | ≤8.0 | 41.2/41.2 | 7-140 | 502/502 | ≤500 | 207/277 | 105-233 | ND | - | ND | - |
| **P14** | 3300/6500 | 1500-5500 | 4100/700/8100 | 1200-7590 | 336/604 | 206-445 | 0.14/31.0 | ≤0.15 | 57/80 | ≤8.0 | 34/40 | 7-140 | 1350/10509 | ≤500 | 417/572 | 160-370 | 0.2/ND | ≤1.0 | 0.26/ND | 0.26-0.61 |
| **P15** | 4110/7310 | 1500-5500 | 2750/1770/4600 | 1200-7590 | 423/423 | 206-445 | 0.19/0.58 | ≤0.15 | 31.2/31.2 | ≤8.0 | 770/770 | 7-140 | 489/55078 | ≤500 | 1090/1090 | 160-370 | 0.47/ND | ≤1.0 | 0.14 | 0.26-0.61 |
| **P16** | 2200/3700 | 1250-3600 | 1400/1400/2100 | 2000-9100 | 187/276 | 135-317 | 0.12/0.17 | ≤0.15 | 53.7/53.7 | ≤8.0 | 295.3/ND | 20-250 | 576/249 | ≤500 | 208/208 | 122-222 | ND/0.49 | ≤1.2 | 0.91/0.97 | 0.74-1.35 |
| **P17** | 1000/1100 | 1250-3600 | 7240/980/31000 | 2000-9100 | ND/876 | 135-317 | 7,3/23.4 | ≤0.15 | 115 /221 | ≤8.0 | 210/441 | 20-250 | 196/ND | ≤500 | 288 /410 | 122-222 | 0.6/ND | ≤1.2 | 0.45/ND | 0.74-1.35 |
| **P18** | 1510/2120 | 1250-3650 | 500/500/1020 | 1900-9310 | 255/ND | 139-320 | ND | - | 7.53/ND | ≤8.0 | ND | - | ND | - | 302.3/ND | 110-283 | ND | - | 0.83/ND | 0.74-1.35 |
| **P19** | 830/2480 | 1450-4450 | 2900/580/2900 | 1700-8050 | 194/328 | 177-381 | 0.1/0.2 | ≤0.15 | 9/32 | ≤8.0 | 104/242 | 7-140 | 40/420 | ≤500 | 213/244 | 105-233 | 0.5/ND | ≤1.2 | 0.8/ND | 0.35-0.86 |
| **P20** | ND | - | ND | - | ND | - | ND | - | ND | - | ND | - | ND | - | ND | - | ND | - | ND | - |
| **P21** | ND | - | ND | - | ND | - | ND | - | ND | - | ND | - | ND | - | ND | - | ND | - | ND | - |
| **P22** | ND | - | ND | - | ND | - | ND | - | ND | - | ND | - | ND | - | ND | - | ND | - | ND | - |

NR, normal range according to age (Hollowell et al., 2005; Mayo Clinic, 2023). PCT, procalcitonine; CRP, C-reactive protein; LDH, lactate dehydrogenase; ND, no data.

Values at admission/highest values are shown, except for neutrophils, that values at admission/lowest values/highest values are shown, when available.

**References**

Hollowell, J.G., O.W. van Assendelft, E.W. Gunter, B.G. Lewis, M. Najjar, and C. Pfeiffer. 2005. Hematological and iron-related analytes-reference data for persons aged 1 year and over: United States, 1988–94. *Vital Health Stat*. 11. 1–156.

Mayo Clinic. 2023. Tests and Procedures - Mayo Clinic. https://www.mayoclinic.org/tests-procedures/index (accessed October 19, 2022)
